# Supplementary material for: Distinct fibroblast subsets regulate lacteal integrity through YAP/TAZ-induced VEGF-C in intestinal villi
Source: Nat Commun. 2020 Aug 14;11:4102. doi: 10.1038/s41467-020-17886-y (PMC7428020; doi:10.1038/s41467-020-17886-y)
Supplement: Supplementary file 1 — Supplementary Information [file 41467_2020_17886_MOESM1_ESM.pdf]

Supplementary Information

# Distinct fibroblast subsets regulate lacteal integrity through YAP/TAZ-induced VEGF-C in intestinal villi

Hong et al.

It includes; Supplementary Figs. 1-17

Supplementary Tables 1 and 2

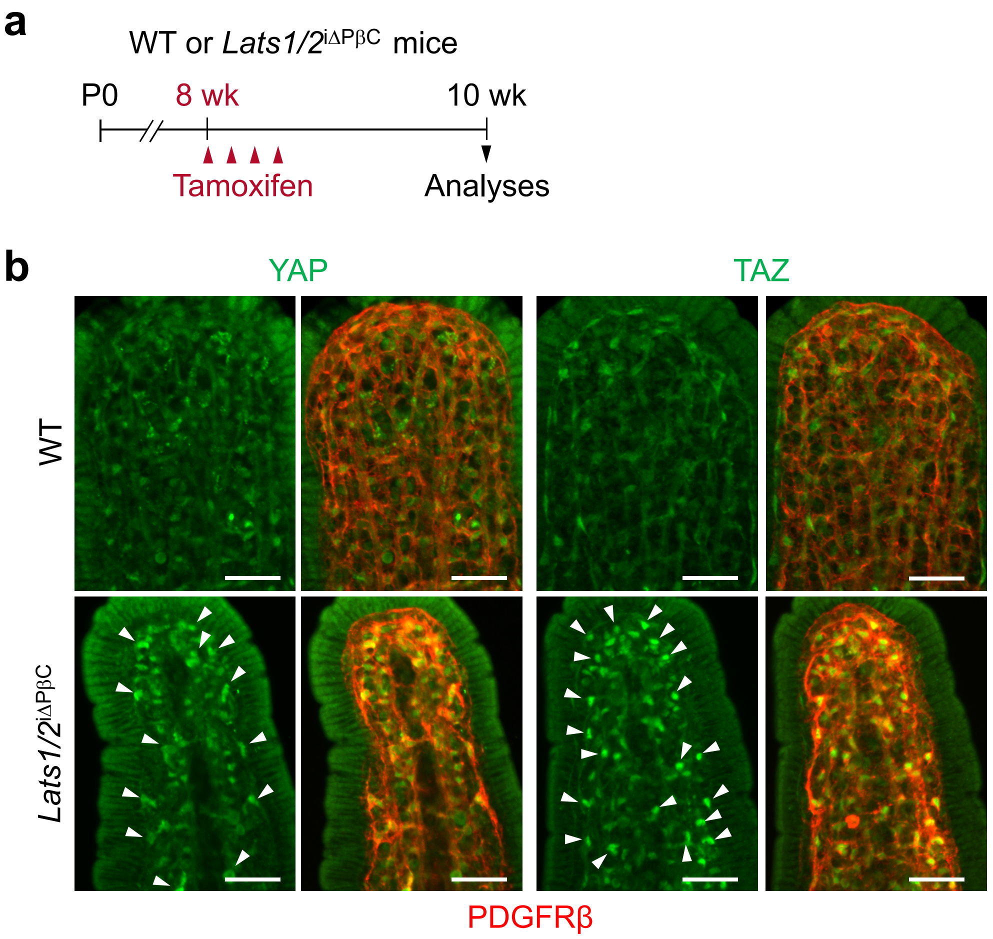


**Supplementary Fig. 1 Efficient activation of YAP/TAZ in IntSCs by *Lats1/2* depletion in PDGFRβ⁺ cells.** **a**, Diagram depicting the PDGFRβ⁺ cell-specific depletion of *Lats1/2* in *Lats1/2*$^{i\Delta P\beta C}$ mice from 8-weeks-old and their analyses at 10-weeks-old. **b**, Representative Images of YAP and TAZ and their subcellular localizations in the PDGFRβ⁺ IntSCs of small intestinal villi in WT and *Lats1/2*$^{i\Delta P\beta C}$ mice. Note that YAP and TAZ are highly localized in the nuclei (white arrowheads) of PDGFRβ⁺ IntSCs in villi of *Lats1/2*$^{i\Delta P\beta C}$ mice. Similar findings were observed in $n$ = 5 mice/group from three independent experiments. Scale bars, 50 μm.

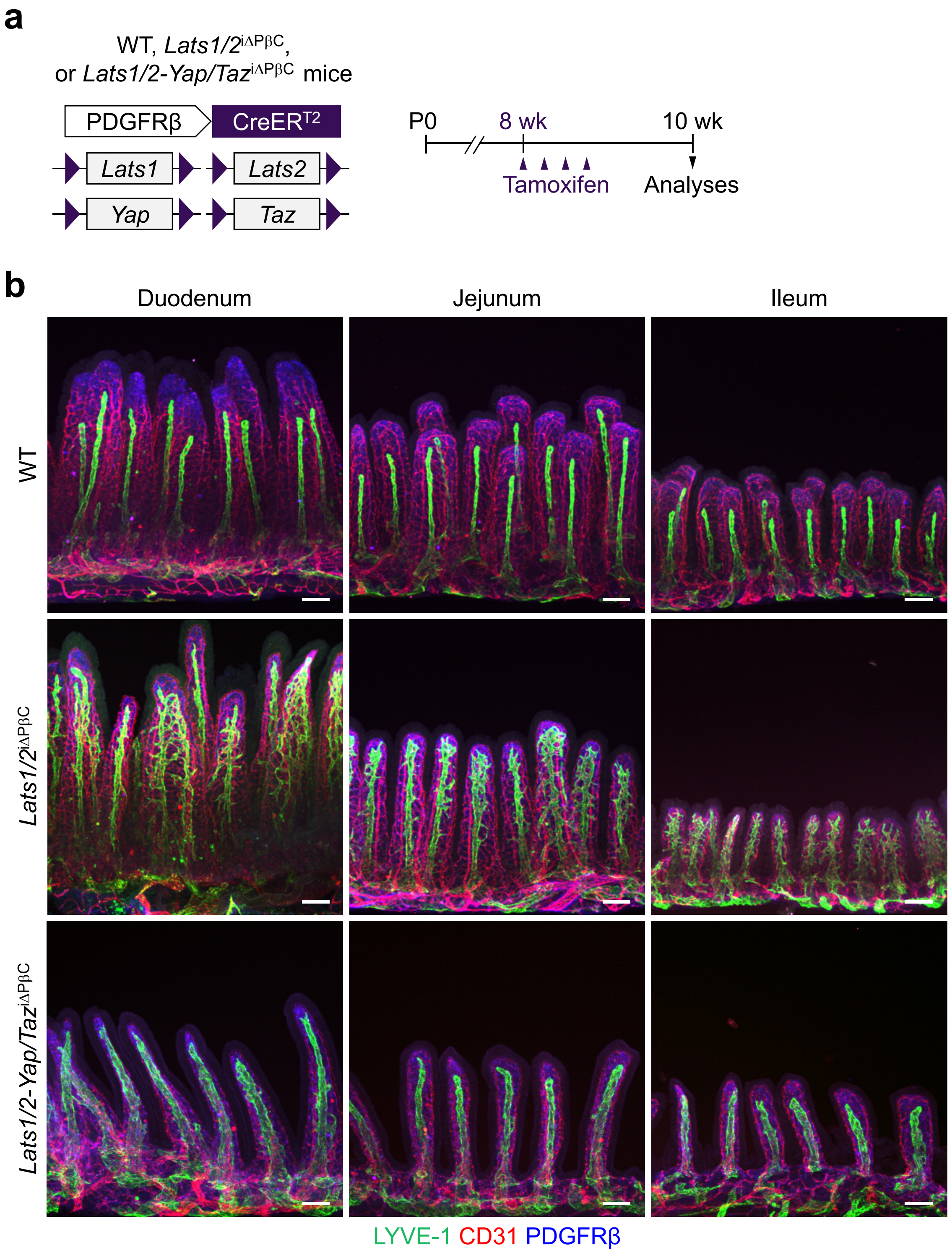


**Supplementary Fig. 2 Rescue of aberrant lacteal phenotypes in *Lats1/2-Yap/Taz*$^{\mathrm{i}\Delta\mathrm{P}\beta\mathrm{C}}$ mice.**

**a**, Diagram depicting the generation of *Lats1/2-Yap/Taz*$^{\mathrm{i}\Delta\mathrm{P}\beta\mathrm{C}}$ mouse and PDGFRβ$^{+}$ cell-specific depletion of *Lats1/2 or Lats1/2-Yap/Taz* by tamoxifen administration in 8-week-old mice and analyses at 2 weeks later. **b**, Representative images of LYVE-1$^{+}$ lacteals, CD31$^{+}$ capillary plexus, and PDGFRβ$^{+}$ stromal cells in duodenum (DD), jejunum (JJ), and ileum (IL) of small intestine in WT, *Lats1/2*$^{\mathrm{i}\Delta\mathrm{P}\beta\mathrm{C}}$, and *Lats1/2-Yap/Taz*$^{\mathrm{i}\Delta\mathrm{P}\beta\mathrm{C}}$ mice. Similar findings were observed in $n$ = 4 mice/group from three independent experiments. Scale bars, 100 μm.

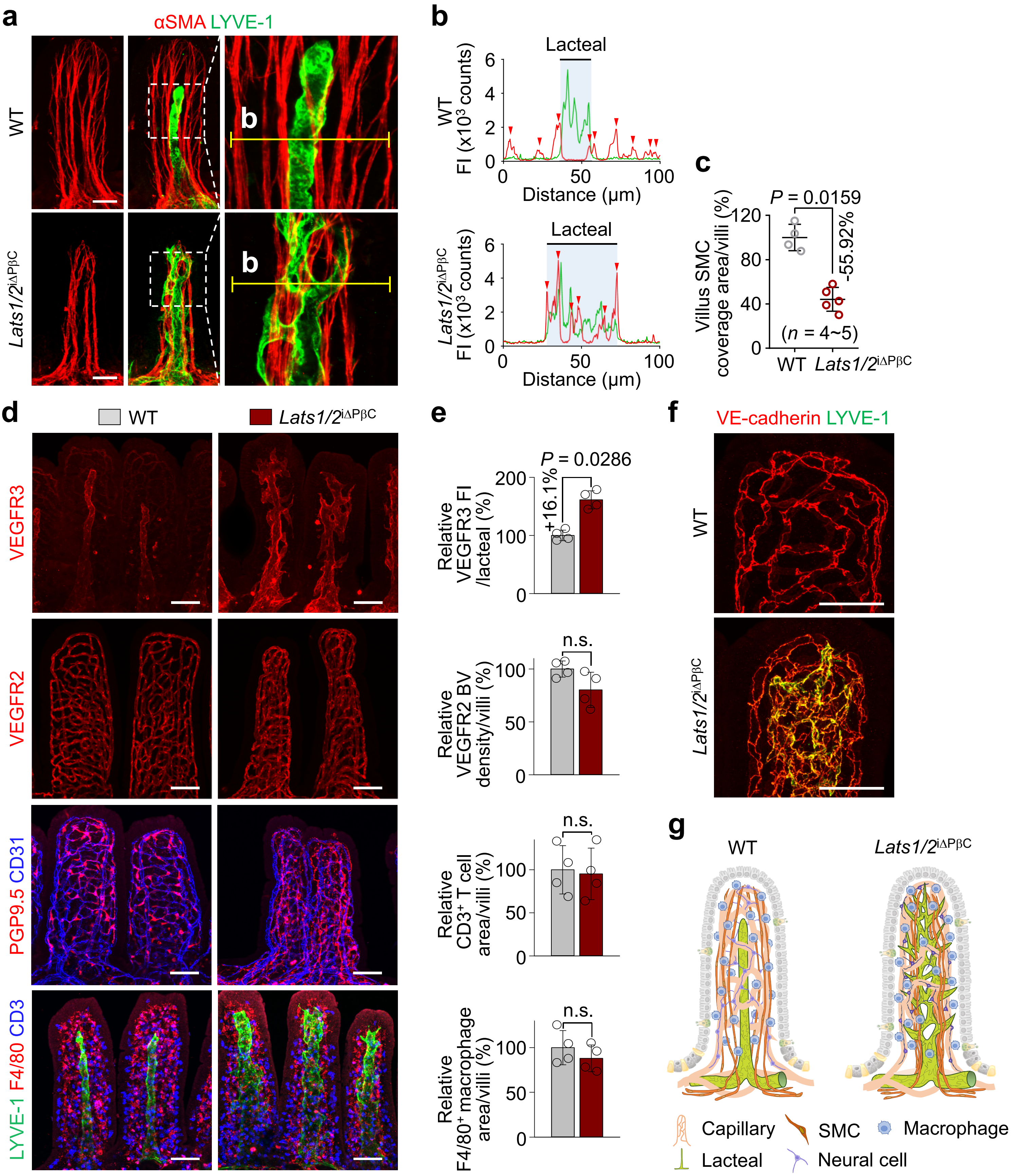


**Supplementary Fig. 3 Skewed alignment of SMCs and enhanced expression of VEGFR3 in *Lats1/2*$^{i\Delta P\beta C}$ mice intestinal villi. a**-**c**, Representative Images and comparison of SMC alignment by profile analysis of the fluorescence intensity (FI) along the indicated yellow lines shown in (**a**) in the villi of WT and *Lats1/2*$^{i\Delta P\beta C}$ mice. Each white dotted-line box is magnified in the right. Note that all the peak αSMA$^{+}$ FI signals (**b**, red arrowheads) of *Lats1/2*$^{i\Delta P\beta C}$ mice are synchronous with the LYVE-1$^{+}$ signal of lacteal and villus SMC coverage is decreased along with its skewed alignment in *Lats1/2*$^{i\Delta P\beta C}$ mice compared with WT. Each dot in indicates a mean value from $n = 4$ (WT) or $n = 5$ (*Lats1/2*$^{i\Delta P\beta C}$) mice pooled from three independent experiments. Horizontal bars indicate mean ± SD. *P* value versus WT by two-tailed Mann-Whitney *U* test. Scale bars, 50 μm.

**d**,**e**, Representative images and comparisons of the intensity of lacteal VEGFR3 expression, VEGFR2$^{+}$ blood vessel (BV) density/villi, distribution of PGP9.5$^{+}$ neural network, and the relative number of CD3$^{+}$ T cell and F4/80$^{+}$ macrophage in the villi of WT and *Lats1/2*$^{i\Delta P\beta C}$ mice. Note that although there are minor changes in capillary plexus density and immune cell number, PGP9.5$^{+}$ neural network along the capillary plexus is disrupted in villi of *Lats1/2*$^{i\Delta P\beta C}$ mice compared with WT. Each dot indicates a mean value obtained from one mouse and $n = 4$ mice/group pooled from three independent experiments. Horizontal bars indicate mean ± SD. *P* value versus WT by two-tailed Mann-Whitney *U* test. n.s., not significant. Scale bars, 50 μm. **f**, Representative images of VE-cadherin$^{+}$ alignment and junctional pattern of the VE-cadherin$^{+}$LYVE-1$^{-}$blood endothelial cells in WT and *Lats1/2*$^{i\Delta P\beta C}$ mice, which show no remarkable differences. Similar findings were observed in $n = 5$ mice/group from three independent experiments. Scale bars, 50 μm. **g**, Schematic images depicting the aberrant sprouting and branching of lacteal, without significant changes in BVs, after YAP/TAZ hyperactivation in PDGFRβ$^{+}$ IntSCs.

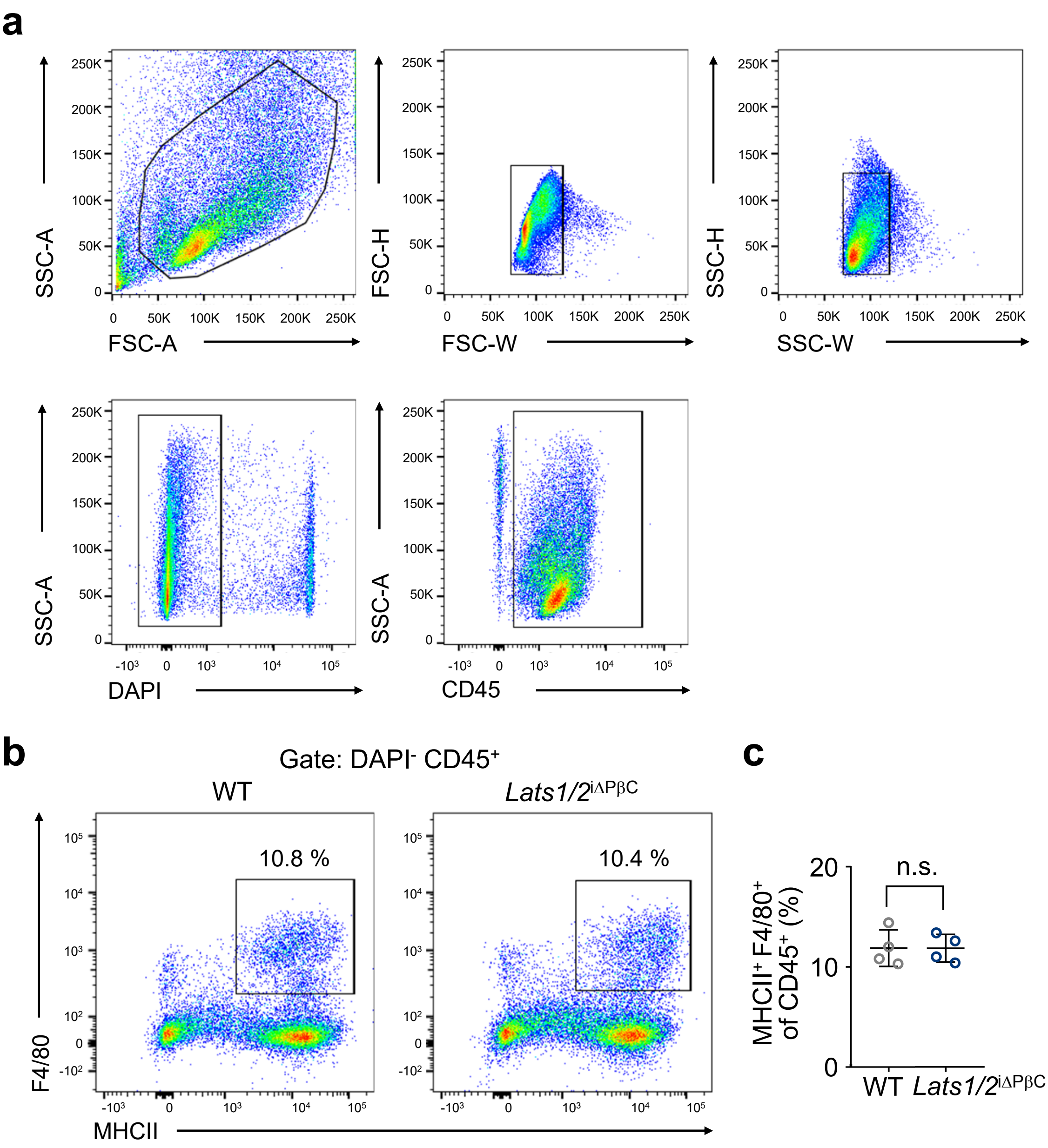


**Supplementary Fig. 4 Flow cytometric analysis of MHCII$^+$ F4/80$^+$ macrophages in WT and *Lats1/2*$^{i\Delta P\beta C}$ mice.**

**a,** Representative flow cytometric analysis with gating strategy from the adult small intestine in WT and *Lats1/2*$^{i\Delta P\beta C}$ mice. **b**,**c**, Representative flow cytometric analysis and comparison of MHCII$^+$ F4/80$^+$ macrophage gated on DAPI$^-$ CD45$^+$ cells from whole small intestine of WT and *Lats1/2*$^{i\Delta P\beta C}$ mice. Each dot indicates value from $n$ = 4 mice/group. Horizontal bars indicate mean ± SD and $P$ value versus WT by two-tailed Mann-Whitney $U$ test. n.s., not significant.

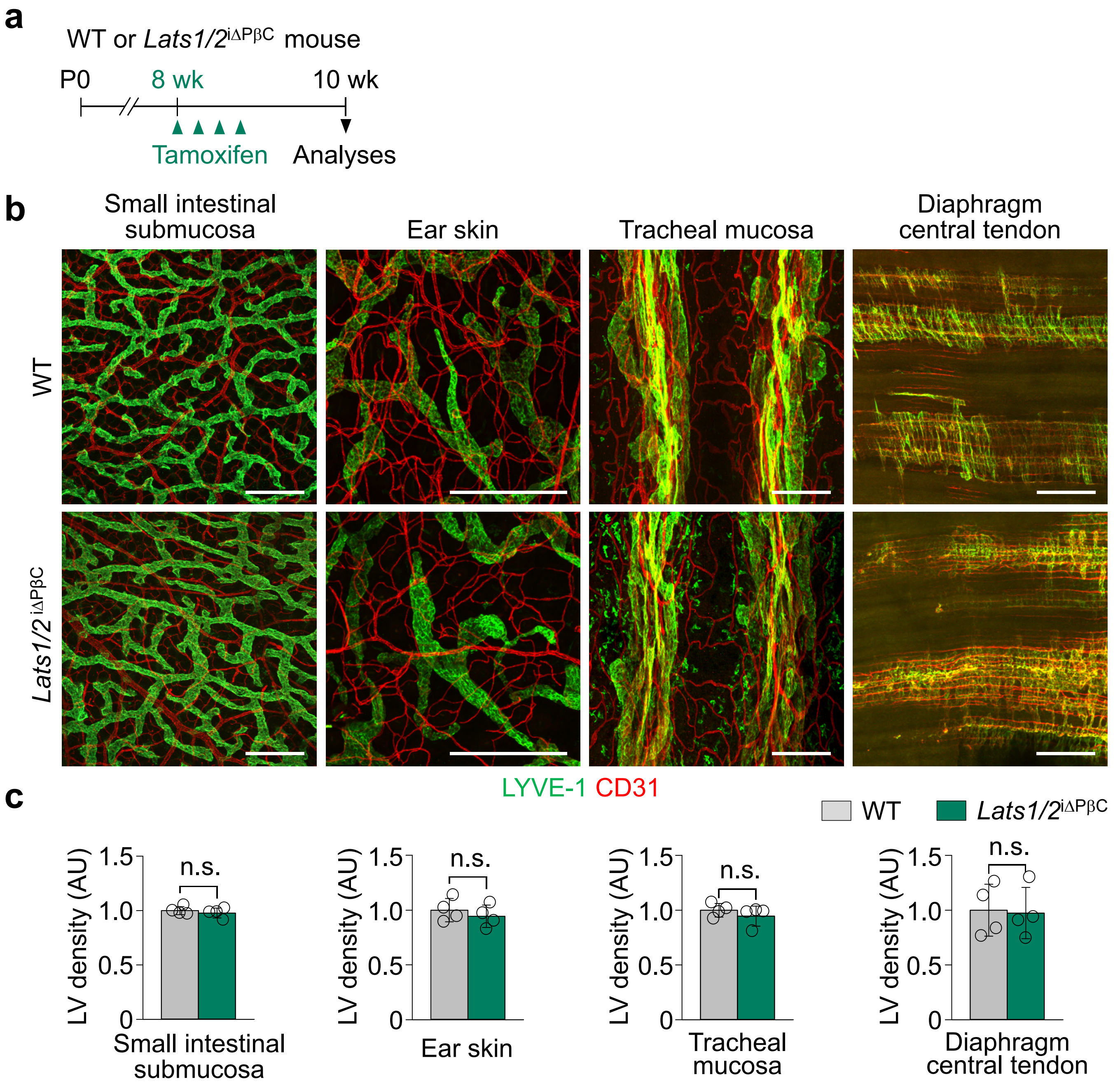


**Supplementary Fig. 5 Minor alterations of LVs in other organs in *Lats1/2*$^{iΔPβC}$ mice.**

**a**, Diagram depicting PDGFRβ$^{+}$ cell-specific depletion of *Lats1/2* in *Lats1/2*$^{iΔPβC}$ mice from 8 weeks and their analyses at 2 weeks later. **b**,**c**, Representative images of LYVE-1$^{+}$ lymphatic vessels (LVs) and comparisons of LV density in small intestinal submucosa, ear skin, tracheal mucosa, and central tendon of diaphragm in WT and *Lats1/2*$^{iΔPβC}$ mice. Each dot indicates a mean value from five different regions of a mouse and $n = 4$ mice/group pooled from three independent experiments. Horizontal bars indicate mean ± SD. *P* value versus WT by two-tailed Mann-Whitney *U* test. AU, arbitrary unit; n.s., not significant. Scale bars, 200 μm.

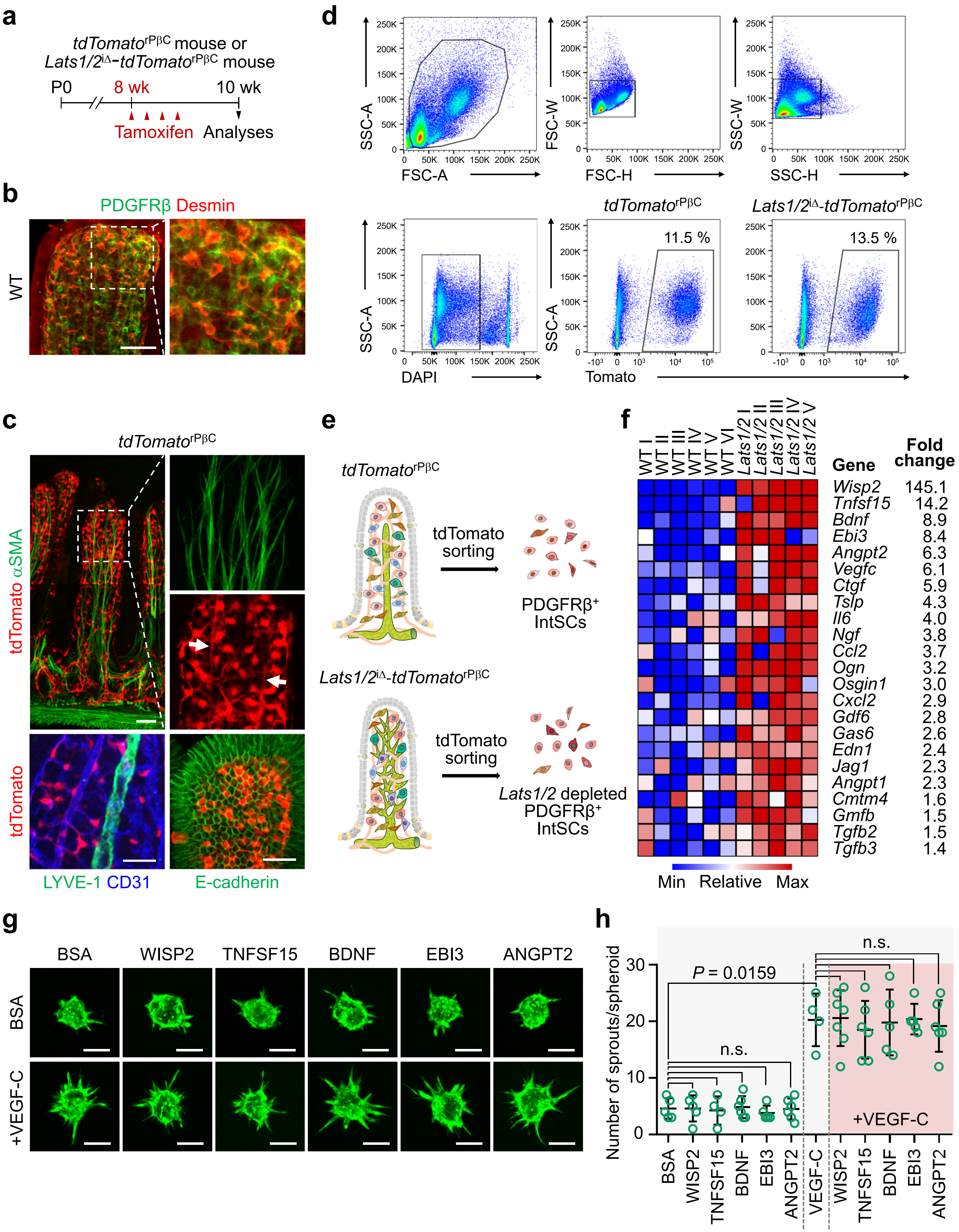
a
tdTomato^rPβC mouse or
Lats1/2^iΔ-tdTomato^rPβC mouse
P0
8 wk
10 wk
Tamoxifen
Analyses
b
PDGFRβ Desmin
WT
c
tdTomato^rPβC
tdTomato αSMA
tdTomato
LYVE-1 CD31
E-cadherin
d
SSC-A
FSC-A
FSC-W
FSC-H
SSC-W
SSC-H
DAPI
Tomato
tdTomato^rPβC
Lats1/2^iΔ-tdTomato^rPβC
11.5 %
13.5 %
e
tdTomato^rPβC
tdTomato sorting
PDGFRβ+ IntSCs
Lats1/2^iΔ-tdTomato^rPβC
tdTomato sorting
Lats1/2 depleted PDGFRβ+ IntSCs
f
WT I
WT II
WT III
WT IV
WT V
WT VI
Lats1/2 I
Lats1/2 II
Lats1/2 III
Lats1/2 IV
Lats1/2 V
Gene
Fold change
Wisp2 145.1
Tnfsf15 14.2
Bdnf 8.9
Ebi3 8.4
Angpt2 6.3
Vegfc 6.1
Ctgf 5.9
Tslp 4.3
Il6 4.0
Ngf 3.8
Ccl2 3.7
Ogn 3.2
Osgin1 3.0
Cxcl2 2.9
Gdf6 2.8
Gas6 2.6
Edn1 2.4
Jag1 2.3
Angpt1 2.3
Cmtm4 1.6
Gmfb 1.5
Tgfb2 1.5
Tgfb3 1.4
Min Relative Max
g
BSA
WISP2
TNFSF15
BDNF
EBI3
ANGPT2
BSA
+VEGF-C
h
Number of sprouts/spheroid
P = 0.0159
n.s.
n.s.
+VEGF-C
BSA
WISP2
TNFSF15
BDNF
EBI3
ANGPT2
VEGF-C
WISP2
TNFSF15
BDNF
EBI3
ANGPT2

**Supplementary Fig. 6 *Vegfc* expression is upregulated in PDGFRβ$^{+}$ IntSCs of *Lats1/2*$^{i\Delta P\beta C}$ mice and is responsible for LEC sprouting.**

**a**, Diagram for PDGFRβ$^{+}$ cell-specific expression of tdTomato in indicated mouse from 8 weeks and their analyses at 10 weeks after birth. **b**,**c**, Representative images of PDGFRβ$^{+}$ IntSCs in the intestinal villi, including desmin$^{+}$ villus fibroblasts (**b**), αSMA$^{+}$ SMCs (**c**, left upper panel, white arrows), pericytes that surround CD31$^{+}$ villus blood vessels (BVs) (**c**, left lower panel) under the E-cadherin$^{+}$ intestinal epithelial cells (**c**, right lower panel). Each white dashed line box is magnified in the right. Similar findings were observed in $n = 3$ (**b**, WT) or $n = 4$ (**c**, *tdTomato*$^{rP\beta C}$) mice from two independent experiments. Scale bars, 50 μm. **d**, Representative flow cytometric analysis with gating strategy and comparisons of Tomato$^{+}$ IntSCs from the adult small intestine in *tdTomato*$^{rP\beta C}$ and *Lats1/2*$^{i\Delta}$-*tdTomato*$^{rP\beta C}$ mice. **e**, Diagram depicting the FACS of Tomato$^{+}$ IntSCs from the adult small intestine in *tdTomato*$^{rP\beta C}$ and *Lats1/2*$^{i\Delta}$-*tdTomato*$^{rP\beta C}$ mice. **f**, Heatmap of the top 23 genes enriched in isolated PDGFRβ$^{+}$ IntSCs from *Lats1/2*$^{i\Delta}$-*tdTomato*$^{rP\beta C}$ mice compared with those of *tdTomato*$^{rP\beta C}$ mice, pooled from three independent experiments using $n = 5\sim6$ mice/group. Note that *Vegfc* is the sixth highest upregulated gene among the others. **g**,**h**, Representative images of LEC spheroids and comparison of the number of sprouts after stimulation with indicated molecules with or without VEGF-C for 24 h. Each dot indicates a value from $n = 4\sim7$ spheroids/group stimulated with the indicated molecule, pooled from four independent experiments. Horizontal bars indicate mean ± SD and $P$ value versus BSA or VEGF-C by two-tailed Mann-Whitney $U$ test. BSA, bovine serum albumin; WISP2, wint1 inducible signaling pathway protein 2; TNF15, tumor necrosis factor ligand superfamily member 15; BDNF, brain-derived neurotrophic factor; EBI3, epstein-barr virus induced 3; ANGPT2, angiopoietin-2; VEGF-C, vascular endothelial growth factor-C; n.s., not significant. Scale bars, 100 μm.

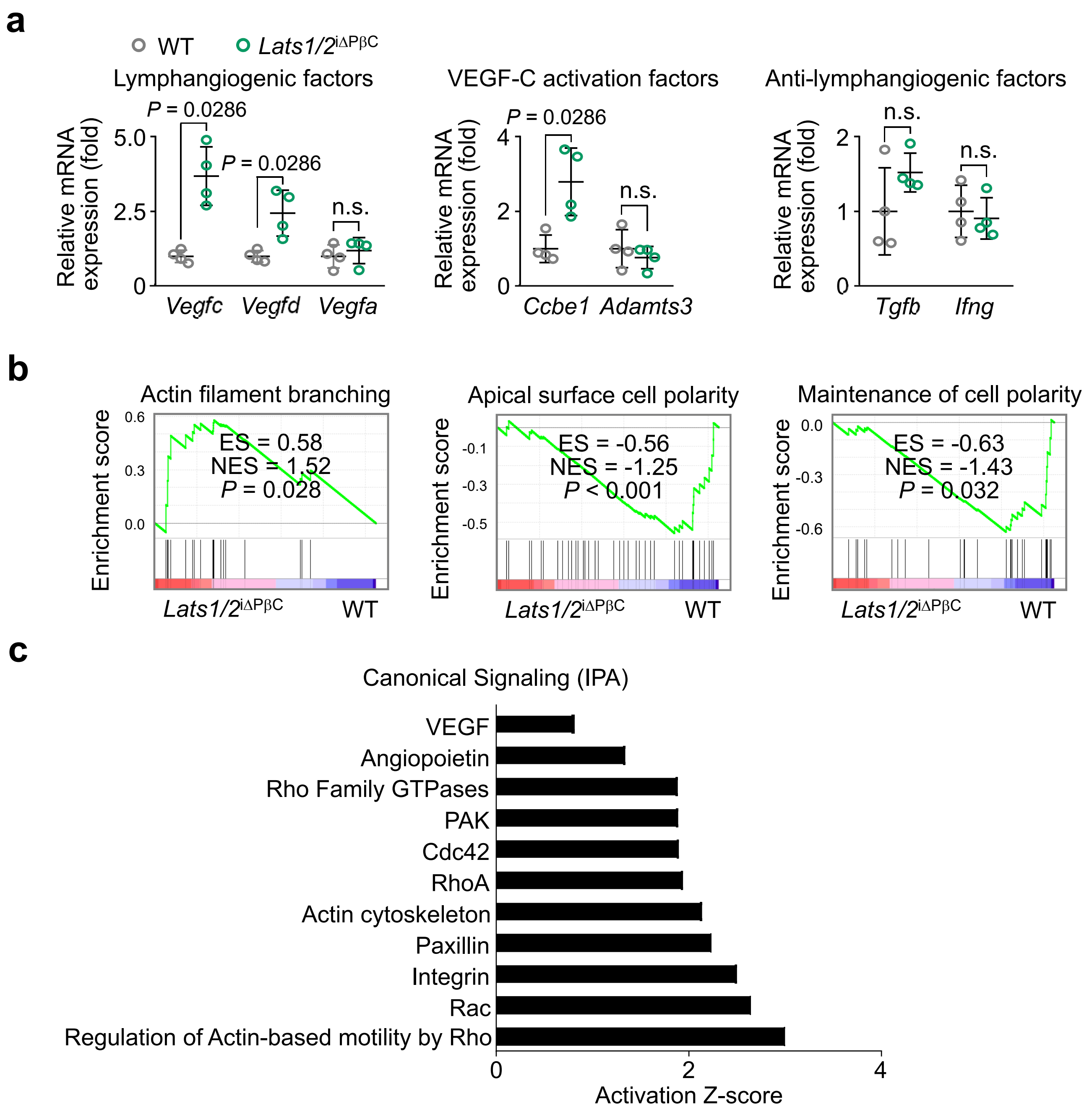


**Supplementary Fig. 7 YAP/TAZ hyperactivation in PDGFRβ⁺ IntSCs promotes *Vegfc*, *Vegfd*, and *Ccbe1* mRNA levels, while attenuating gene sets related to vessel integrity in lacteal LECs.**

**a**, Comparison of indicated mRNA expressions in intestinal villi lysates of WT and *Lats1/2*$^{i\Delta P\beta C}$ mice. Data from $n = 4$ mice/group pooled from two independent experiments. Horizontal bars indicate mean ± SD and $P$ value versus WT by two-tailed Mann-Whitney $U$ test. n.s., not significant. **b**, Gene set enrichment analysis of differentially expressed genes in isolated intestinal LECs from the adult small intestinal villi lysates of *tdTomato*$^{rP\beta C}$ and *Lats1/2*$^{i\Delta}$*tdTomato*$^{rP\beta C}$ mice. $n$ = 3~4 mice/group pooled from two independent experiments. ES, enrichment score; NES, normalized enrichment score. **c**, Ingenuity Pathway Analysis (IPA) microarray of transcriptome changes in canonical signaling pathway after YAP/TAZ hyperactivation in intestinal LECs of WT and *Lats1/2*$^{i\Delta P\beta C}$ mice. Note the transcript changes ascertaining their status in sorted intestinal LECs of *Lats1/2*$^{i\Delta}$*tdTomato*$^{rP\beta C}$ mice compared with *tdTomato*$^{rP\beta C}$ mice.

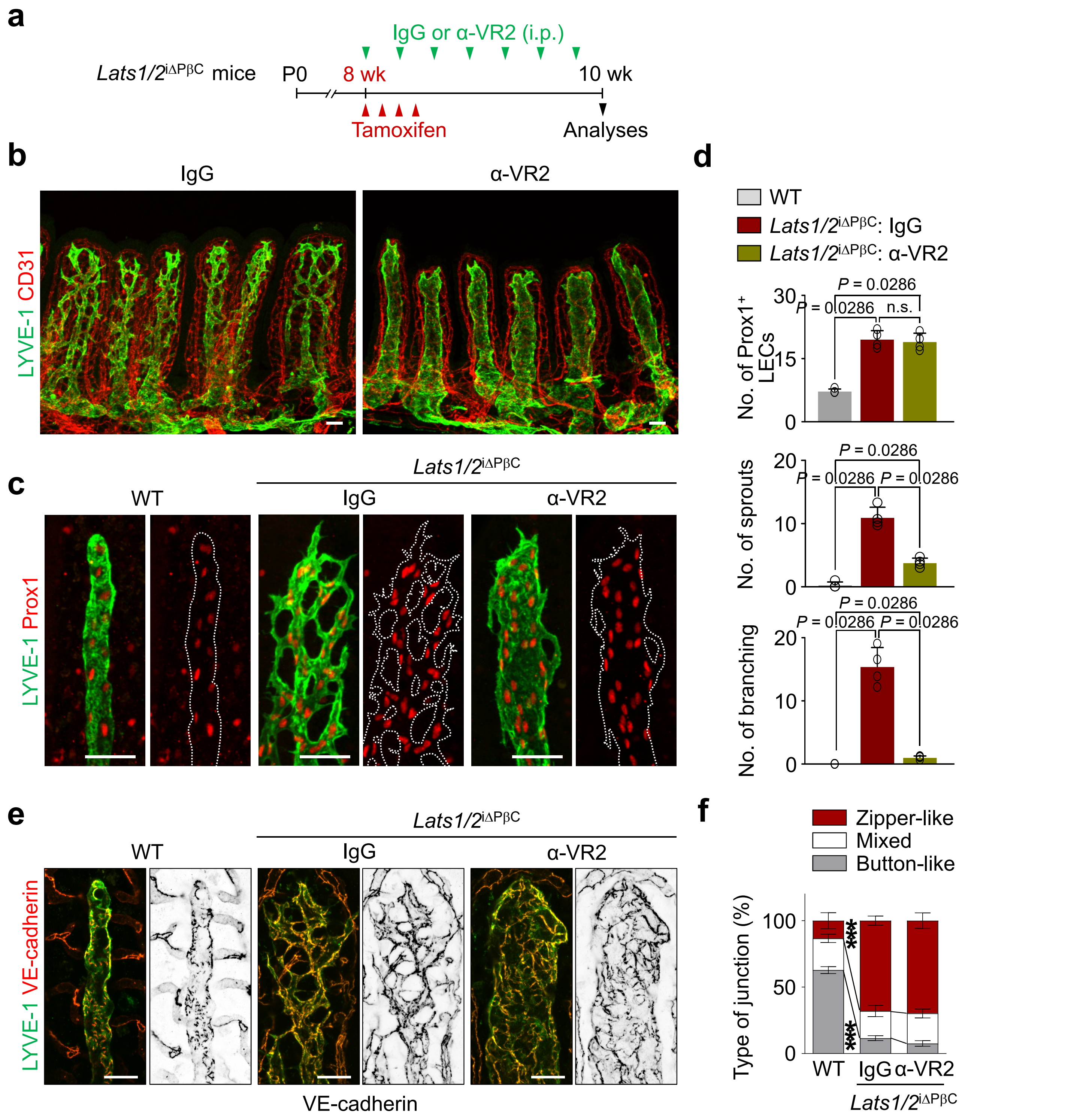


**Supplementary Fig. 8 VEGFR2 blockade partially alleviates the lacteal hypersprouting and branching in *Lats1/2*$^{i\Delta P\beta C}$ mice. a**, Diagram for PDGFRβ$^{+}$ cell-specific depletion of *Lats1/2* in *Lats1/2*$^{i\Delta P\beta C}$ mice by tamoxifen delivery at 8-weeks-old, and intraperitoneal (i.p.) injection of IgG-Fc (IgG, control) or α-VEGFR2 (α-VR2) for two weeks prior to analyses. **b**-**d**, Representative images of CD31$^{+}$ blood capillary plexus and LYVE-1$^{+}$ lacteals after treatment with IgG or α-VR2 in *Lats1/2*$^{i\Delta P\beta C}$ mice and comparisons of the number of Prox1$^{+}$ lymphatic endothelial cells (LECs), lacteal sprouts, and lacteal branches per 100 μm of lacteal length in WT and *Lats1/2*$^{i\Delta P\beta C}$ mice after IgG or α-VR2 treatment. Each dot indicates a mean value of 10-20 villi/mouse and $n = 4$ mice/group pooled from two independent experiments. Horizontal bars indicate mean ± SD and *P* value versus WT or IgG by two-tailed Mann-Whitney *U* test. n.s., not significant. Scale bars, 50 μm. **e**,**f**, Representative images and comparison of VE-cadherin$^{+}$ LEC junctions of LYVE-1$^{+}$ lacteals in the villi of WT and *Lats1/2*$^{i\Delta P\beta C}$ mice after IgG or α-VR2 treatments. Horizontal bars of each colored segment represent means ± SD of 5-10 villi/mouse and $n = 4$ mice/group pooled from two independent experiments. *** $P < 0.0001$ versus WT by two-way ANOVA with Holm-Sidak's multiple comparisons test. n.s., not significant. Scale bars, 25 μm.

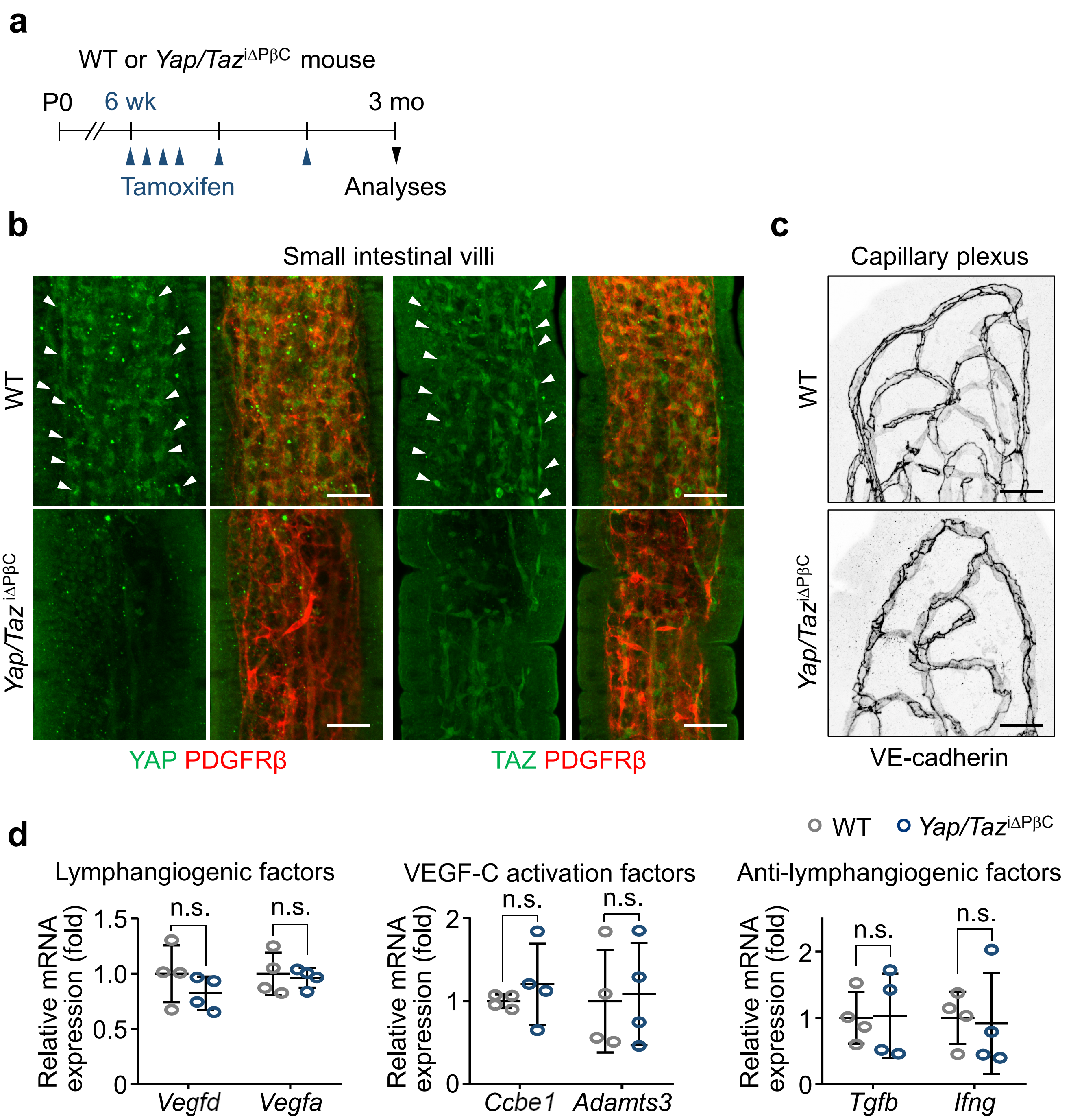


**Supplementary Fig. 9 Minor alterations in junctional pattern of capillary plexus and lymphangiogenesis-related transcripts other than *Vegfc* in intestinal villi of *Yap/Taz*$^{i\Delta P\beta C}$ mice.**

**a**, Diagram for PDGFRβ$^{+}$ cell-specific depletion of *Yap/Taz* in *Yap/Taz*$^{i\Delta P\beta C}$ mice from 6 week and their analyses at 3 months after birth. **b**, Representative images of YAP (left panels, white arrowheads) and TAZ (right panels, white arrowheads) in the small intestinal villi of WT and *Yap/Taz*$^{i\Delta P\beta C}$ mice. Similar findings were observed in $n$ = 5 mice/group from two independent experiments. Scale bars, 50 μm. **c**, Representative images of VE-cadherin$^{+}$ blood endothelial cell junctions of capillary plexus in the small intestinal villi of WT and *Yap/Taz*$^{i\Delta P\beta C}$ mice. Similar findings were observed in $n$ = 5 mice/group from two independent experiments. Scale bars, 25 μm. **d**, Comparisons of indicated mRNA expressions in intestinal villi lysates of WT and *Yap/Taz*$^{i\Delta P\beta C}$ mice. Data of $n$ = 4 mice/group pooled from two independent experiments. Horizontal bars indicate mean ± SD. *P* value versus WT by two-tailed Mann-Whitney *U* test. n.s., not significant.

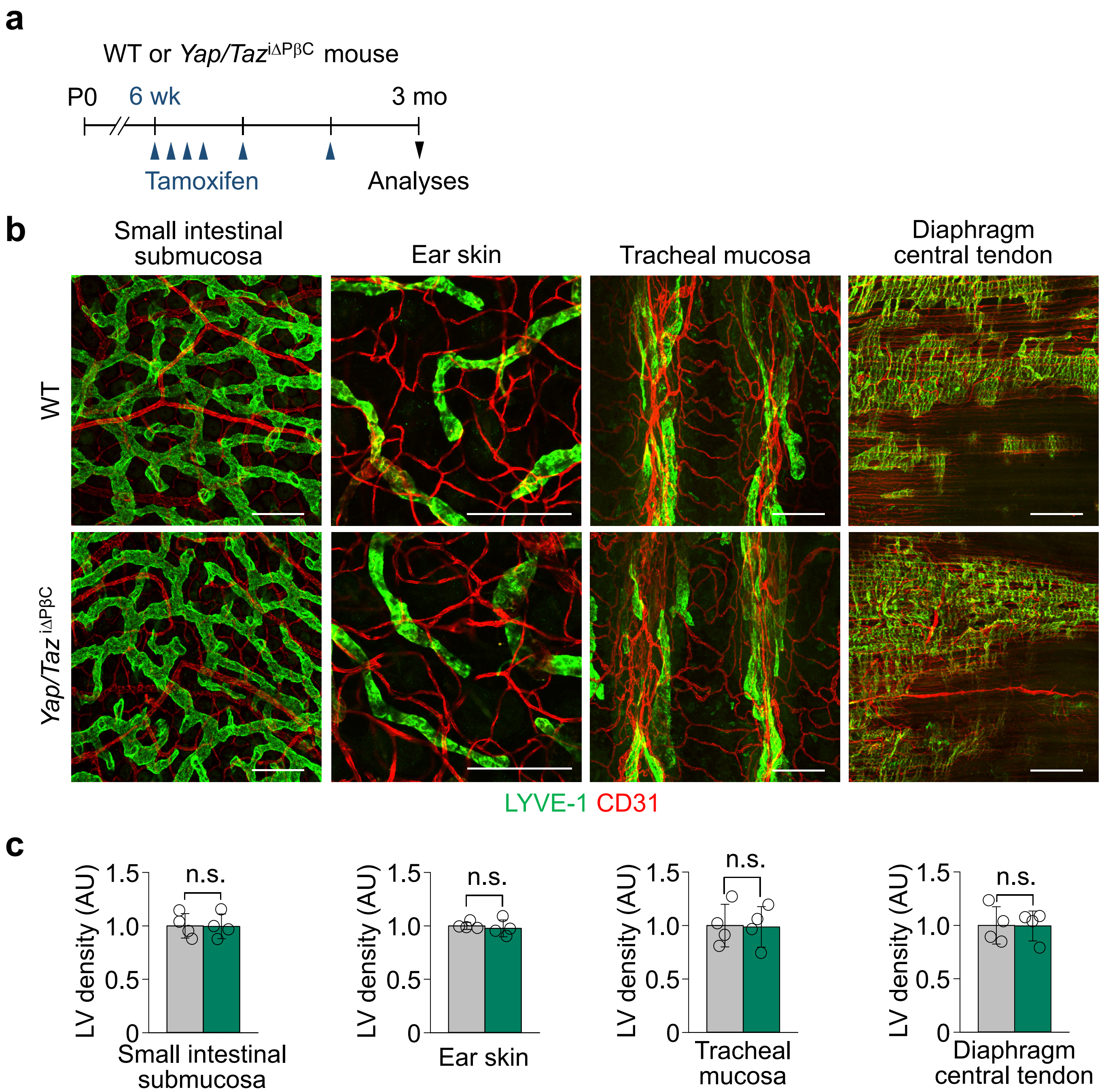


**Supplementary Fig. 10 Minor alterations of other organ LVs in *Yap/Taz*$^{i\Delta P\beta C}$ mice.**

**a**, Diagram depicting PDGFRβ$^{+}$ cell-specific depletion of *Yap/Taz* from 6 week and their analyses at 3 months after birth. **b**,**c**, Representative images of LYVE-1$^{+}$ lymphatic vessels (LVs) and comparisons of LV density in small intestinal submucosa, ear skin, tracheal mucosa, and central tendon of diaphragm in WT and *Yap/Taz*$^{i\Delta P\beta C}$ mice. Each dot indicates a mean value of five different regions of a mouse and $n = 4$ mice/group pooled from three independent experiments. Horizontal bars indicate mean ± SD. *P* value versus WT by two-tailed Mann-Whitney *U* test. AU, arbitrary unit; n.s., not significant. Scale bars, 200 μm.

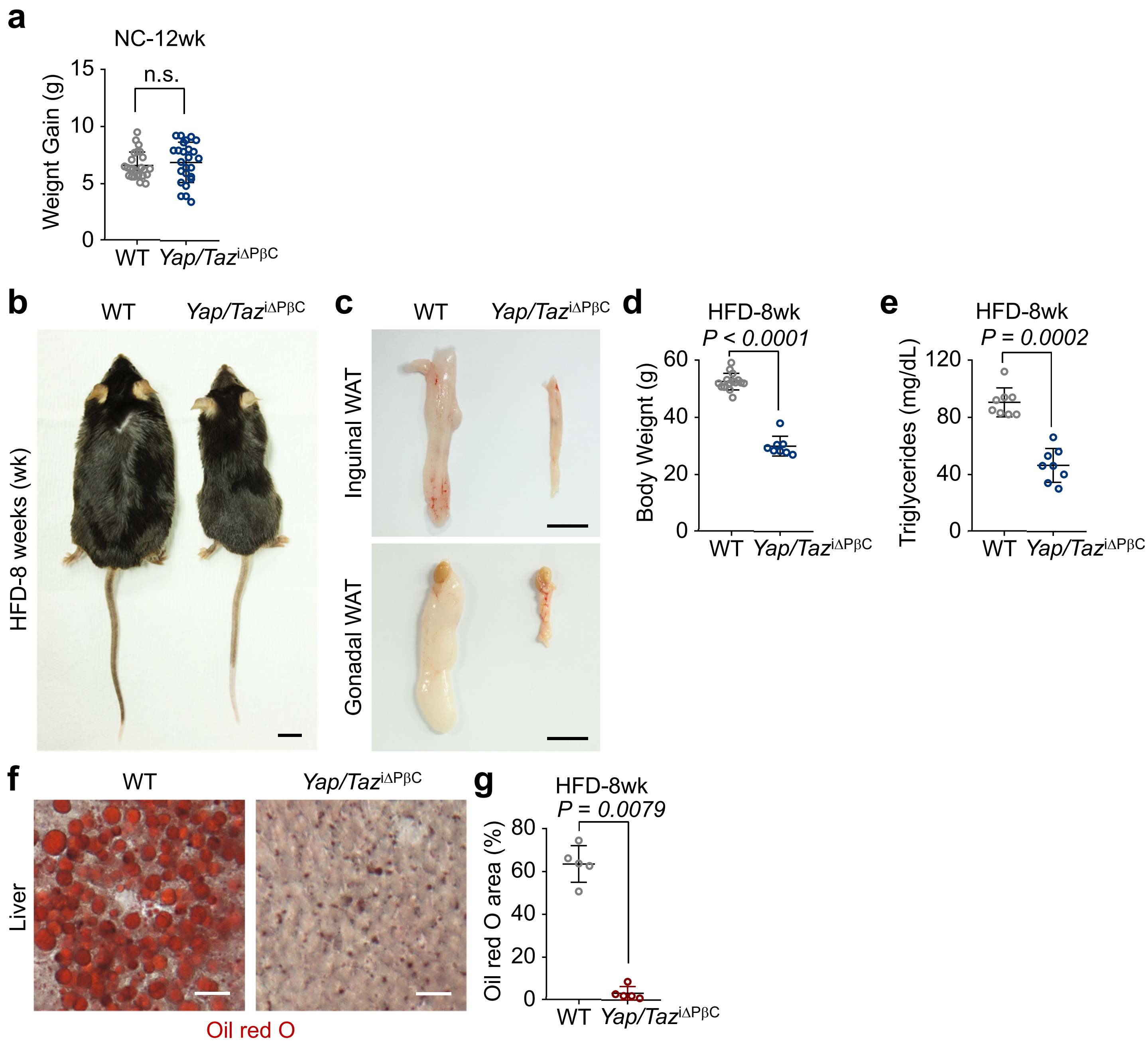


**Supplementary Fig. 11 Impaired dietary lipid absorption in *Yap/Taz*$^{i\Delta P\beta C}$ mice.**

**a**, Comparison of weight gain of normal chow (NC)-fed WT and *Yap/Taz*$^{i\Delta P\beta C}$ mice. Each dot indicates a value from $n$ = 25 mice/group. Horizontal bars indicate mean ± SD and $P$ value versus WT by two-tailed Mann-Whitney $U$ test. n.s., not significant. **b**, Gross morphology of WT and *Yap/Taz*$^{i\Delta P\beta C}$ mice after 8 weeks of high-fat diet (HFD). Scale bars, 1 cm. **c**, Representative gross image of inguinal white adipose tissue (WAT) and gonadal WAT of WT and *Yap/Taz*$^{i\Delta P\beta C}$ mice after 8 weeks of HFD. Scale bars, 1 cm. **d**, Comparison of body weight of WT and *Yap/Taz*$^{i\Delta P\beta C}$ mice after 8 weeks of HFD. Each dot indicates a value from $n$ = 8 to 15 mice/group. Horizontal bars indicate mean ± SD and $P$ < 0.0001 versus WT by two-tailed Mann-Whitney $U$ test. **e**, Comparison of plasma triglyceride concentration (mg/dL) in 6 hours fasted WT and *Yap/Taz*$^{i\Delta P\beta C}$ mice after 8 weeks of HFD. Each dot indicates a value from $n$ = 8 mice/group. Horizontal bars indicate mean ± SD and $P$ value versus WT by two-tailed Mann-Whitney $U$ test. **f**,**g**, Representative image and comparison of Oil red O staining in liver of WT and *Yap/Taz*$^{i\Delta P\beta C}$ mice after 8 weeks of HFD. Each dot indicates a mean value taken from five different regions of a mouse and $n$ = 5 mice/group. Horizontal bars indicate mean ± SD and $P$ value versus WT by two-tailed Mann-Whitney $U$ test. Scale bars, 50 μm.

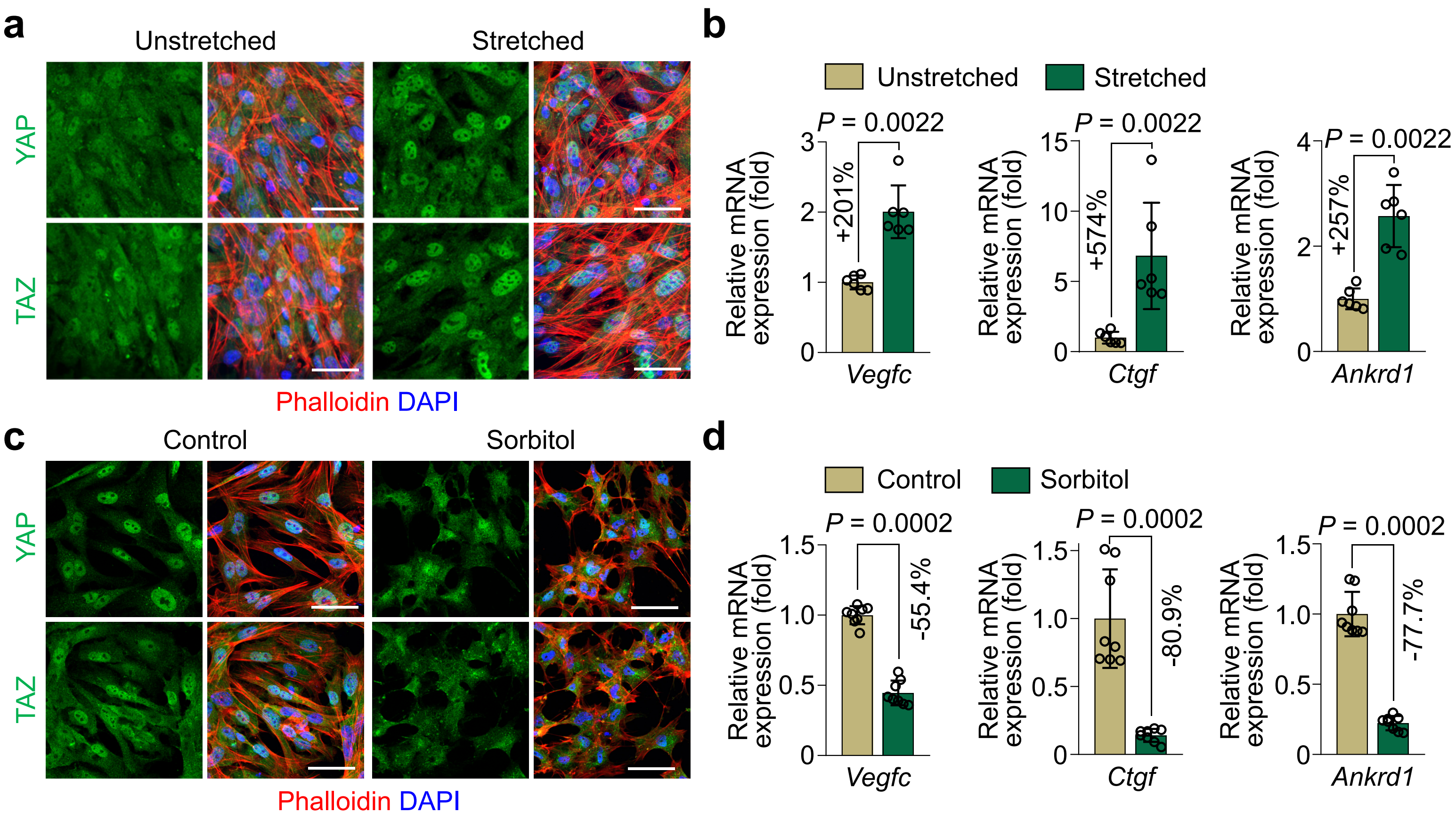


**Supplementary Fig. 12 Mechanical and osmotic stress regulate YAP/TAZ activation and *Vegfc* in fibroblasts.**

**a**, Representative images of YAP and TAZ with phalloidin⁺ actin filaments in unstretched and stretched (4% of linear stretch at 10 cycles/min for 3h) MEFs with an automated cell-stretching system (STREX). Note the enhancements of nuclear YAP and TAZ after mechanical stretch in MEFs. Similar results were observed in four independent experiments. Scale bars, 50 μm. **b**, Comparison of mRNA expressions of *Vegfc*, *Ctgf* and *Ankrd1* after mechanical stretch in MEFs. Dots indicate data from six independent experiments and horizontal bars indicate mean ± SD. *P* value versus Unstretched by two-tailed Mann-Whitney *U* test. **c,** Representative images of YAP and TAZ with phalloidin⁺ actin filaments in MEFs with or without 0.4 M sorbitol-induced osmotic stress for 3 h. Note the cytoplasmic translocation of YAP and TAZ after the osmotic stress in MEFs. Similar results were observed in four independent experiments. Scale bars, 50 μm. **d**, Comparison of mRNA expressions of *Vegfc*, *Ctgf* and *Ankrd1* after osmotic stress in MEFs. Dots indicate data from eight independent experiments and horizontal bars indicate mean ± SD. *P* value versus Control by two-tailed Mann-Whitney *U* test.

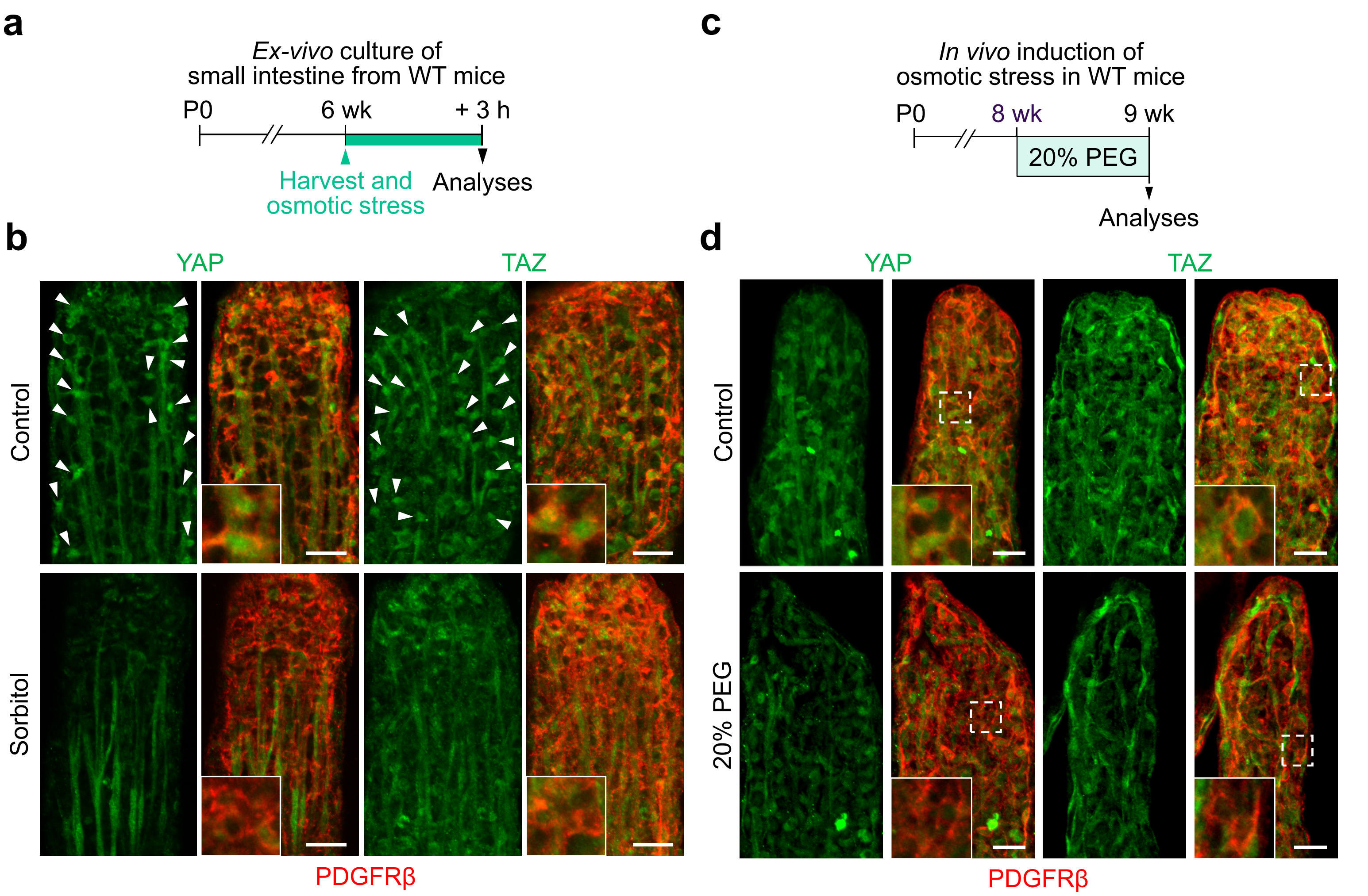


**Supplementary Fig. 13 Osmotic stress regulates translocation of YAP/TAZ in PDGFRβ⁺ IntSCs *ex vivo* and *in vivo*.**

**a**, Diagram for applying osmotic stress (0.4 M sorbitol) into the isolated small intestine from WT mice for the analysis after 3 hours. **b**, Representative images and comparisons of YAP and TAZ expressions and their localizations in the IntSCs of control (white arrowheads indicate YAP or TAZ nucleocytoplasmic localizations) or osmotic stress applied isolated mouse small intestine *ex vivo*. Insets show magnified view of the indicated region. Similar findings were observed in four independent experiments using *n* = 4 mice. Scale bars, 25 μm. **c**, Diagram for inducing osmotic stress in small intestine of WT mice by administration of 20% PEG starting at 8 weeks for 7 days and their analyses at 9 weeks. **d**, Representative images and comparisons of YAP and TAZ expression and their localizations in PDGFRβ⁺ IntSCs of control or osmotic stress applied small intestine *in vivo*. Insets show magnified view. Similar findings were observed in four independent experiments using *n* = 4 mice. Scale bars, 25 μm.

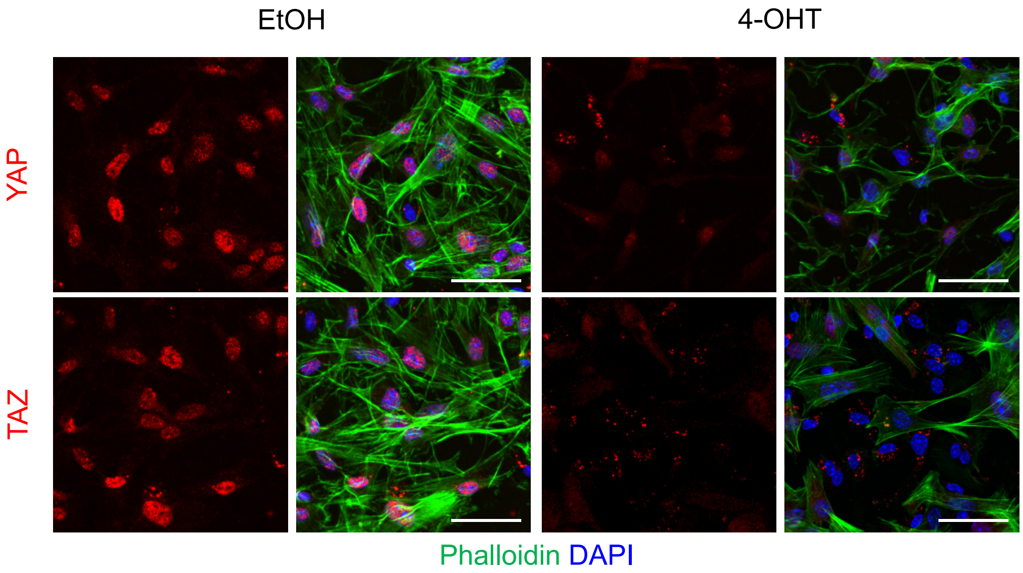


**Supplementary Fig. 14 Administration of 4-OHT depletes YAP/TAZ in IntSCs of *Yap/Taz*$^{i\Delta P\beta C}$ mice.**

Representative images of YAP and TAZ with phalloidin$^{+}$ actin filaments in isolated IntSCs from *Yap/Taz*$^{i\Delta P\beta C}$ mice at 2 days after the EtOH or 4-OHT treatment. Similar findings were observed in four independent experiments. Scale bars, 50 μm.

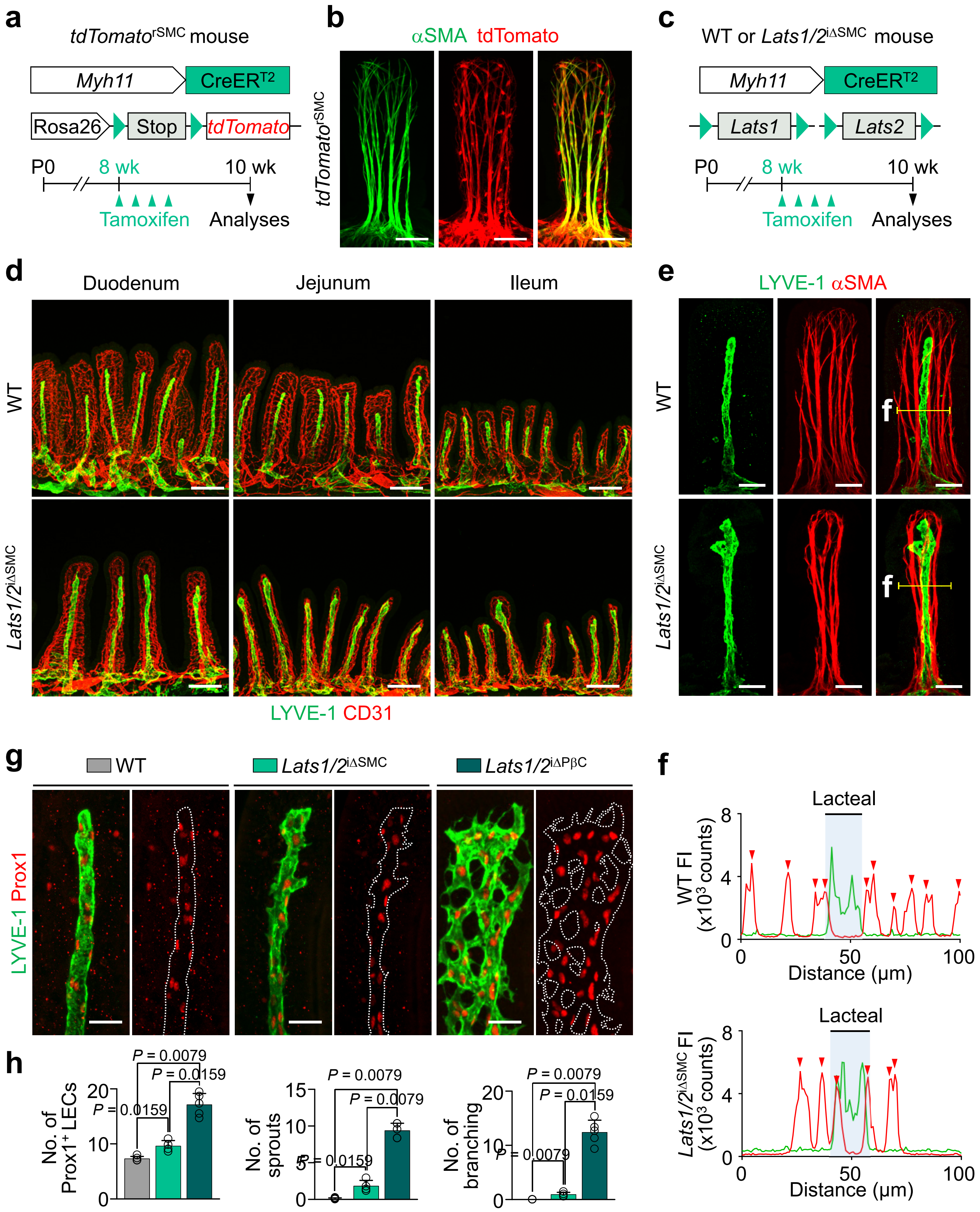


**Supplementary Fig. 15 Selective YAP/TAZ hyperactivation in villus SMCs does not recapitulate the lacteal phenotype of *Lats1/2*$^{i\Delta P\beta C}$ mice. a**, Diagram depicting the generation of *tdTomato*$^{rSMC}$ mouse and SMC-specific expression of Tomato in small intestinal villi from 8 weeks and their analyses at 10 weeks after birth. **b**, Representative images of Tomato$^{+}$ expression in the small intestine of *tdTomato*$^{rSMC}$ mouse, which outlines its relatively selective distribution in the longitudinal αSMA$^{+}$ SMCs and undefined stromal cells. Similar findings were observed in *n* = 4 mice from two independent experiments. Scale bars, 50 μm. **c**, Diagram depicting the generation of *Lats1/2*$^{i\Delta SMC}$ mouse and Myh11$^{+}$ cell specific depletion of *Lats1/2* in small intestine from 8 weeks and their analyses at 10 weeks after birth.

**d**, Representative images and comparisons of LYVE-1$^+$ lacteals and CD31$^+$ blood capillary plexus in the villi of indicated parts of small intestine in WT and *Lats1/2*$^{i\Delta SMC}$ mice. Note that lacteals of *Lats1/2*$^{i\Delta SMC}$ mice do not phenocopy those of *Lats1/2*$^{i\Delta P\beta C}$ mice. Similar findings were observed in *n* = 4 mice/group from two independent experiments. Scale bars, 100 μm. **e**,**f**, Representative images and comparison of villus αSMA$^+$ cells by profile analysis of the fluorescence intensity (FI) along the indicated yellow lines shown in (**e**) in WT and *Lats1/2*$^{i\Delta SMC}$ mice. Note that all the peak αSMA FI signals (red arrowheads) of *Lats1/2*$^{i\Delta SMC}$ are more gathered toward the lacteal and the area of villus SMC coverage is decreased along with its skewed alignment in *Lats1/2*$^{i\Delta SMC}$ mice compared with WT. Similar findings were observed in *n* = 4 mice/group from two independent experiments. Scale bars, 50 μm. **g**,**h**, Representative images and comparisons of the numbers of Prox1$^+$ lymphatic endothelial cells (LECs), lacteal sprouts and lymphatic branches per 100 μm of lacteal length in WT, *Lats1/2*$^{i\Delta SMC}$ and *Lats1/2*$^{i\Delta P\beta C}$ mice. Each dot indicates a mean value of 5 villi/mouse and *n* = 4 mice/group except for *Lats1/2*$^{i\Delta P\beta C}$ (*n* = 5 mice) pooled from two independent experiments. Horizontal bars indicate mean ± SD and *P* value versus WT by two-tailed Mann-Whitney *U* test. Scale bars, 25 μm.

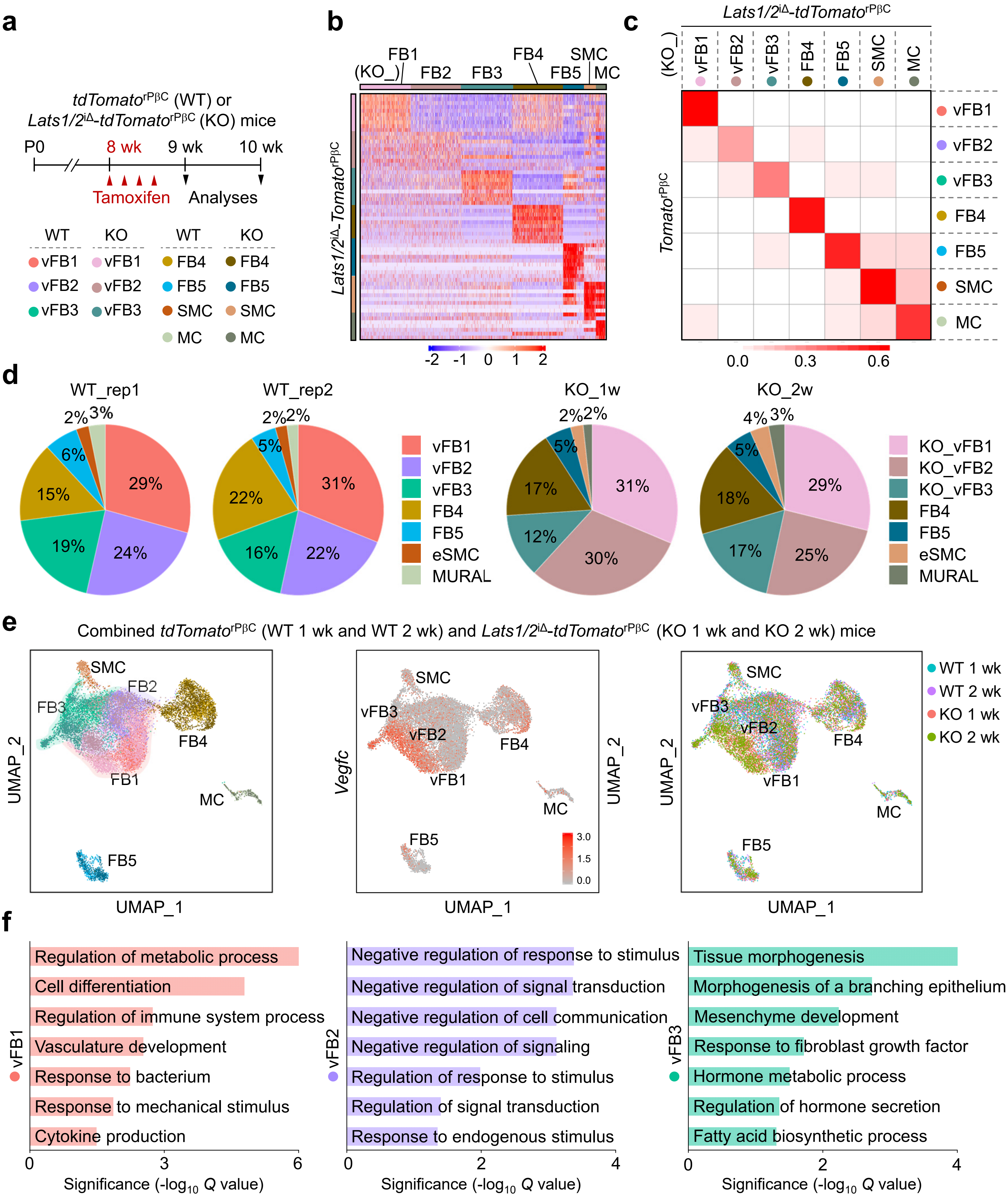

a
tdTomato^rPβC (WT) or
Lats1/2^iΔ-tdTomato^rPβC (KO) mice
P0
8 wk
9 wk
10 wk
Tamoxifen
Analyses
WT
KO
WT
KO
vFB1
vFB1
FB4
FB4
vFB2
vFB2
FB5
FB5
vFB3
vFB3
SMC
SMC
MC
MC
b
FB1
(KO_)
FB2
FB3
FB4
FB5
SMC
MC
Lats1/2^iΔ-Tomato^rPβC
-2 -1 0 1 2
c
Lats1/2^iΔ-tdTomato^rPβC
(KO_)
vFB1
vFB2
vFB3
FB4
FB5
SMC
MC
Tomato^rPβC
vFB1
vFB2
vFB3
FB4
FB5
SMC
MC
0.0
0.3
0.6
d
WT_rep1
2%
3%
6%
15%
29%
19%
24%
WT_rep2
2%
2%
5%
22%
31%
16%
22%
vFB1
vFB2
vFB3
FB4
FB5
eSMC
MURAL
KO_1w
2%
2%
5%
17%
31%
12%
30%
KO_2w
4%
3%
5%
18%
29%
17%
25%
KO_vFB1
KO_vFB2
KO_vFB3
FB4
FB5
eSMC
MURAL
e
Combined tdTomato^rPβC (WT 1 wk and WT 2 wk) and Lats1/2^iΔ-tdTomato^rPβC (KO 1 wk and KO 2 wk) mice
SMC
FB2
FB3
FB4
FB1
MC
FB5
UMAP_2
UMAP_1
SMC
vFB3
vFB2
FB4
vFB1
MC
FB5
Vegfc
3.0
1.5
0.0
UMAP_1
UMAP_2
SMC
vFB3
vFB2
FB4
vFB1
MC
FB5
UMAP_2
UMAP_1
WT 1 wk
WT 2 wk
KO 1 wk
KO 2 wk
f
vFB1
Regulation of metabolic process
Cell differentiation
Regulation of immune system process
Vasculature development
Response to bacterium
Response to mechanical stimulus
Cytokine production
0
3
6
Significance (-log10 Q value)
vFB2
Negative regulation of response to stimulus
Negative regulation of signal transduction
Negative regulation of cell communication
Negative regulation of signaling
Regulation of response to stimulus
Regulation of signal transduction
Response to endogenous stimulus
0
2
4
Significance (-log10 Q value)
vFB3
Tissue morphogenesis
Morphogenesis of a branching epithelium
Mesenchyme development
Response to fibroblast growth factor
Hormone metabolic process
Regulation of hormone secretion
Fatty acid biosynthetic process
0
2
4
Significance (-log10 Q value)

**Supplementary Fig. 16 Unsupervised clustering on the integrated dataset of PDGFRβ$^+$ IntSCs from *tdTomato*$^{rPβC}$ and *Lats1/2*$^{iΔ}$-*tdTomato*$^{rPβC}$ mice suggests that vFB1-3 secrete VEGF-C.**

**a**, Diagram depicting PDGFRβ$^+$ cell-specific expression of tdTomato from 8 weeks and their analyses at 9 weeks or 10 weeks after birth in *tdTomato*$^{rPβC}$ or *Lats1/2*$^{iΔ}$-*tdTomato*$^{rPβC}$ mouse. vFB, intestinal villi fibroblast; FB, fibroblast; SMC, smooth muscle cell; MC, mural cell. **b**, Heatmap displaying the scaled expression patterns of top ten differentially expressed genes for random sampled cells (maximum thousand cells) for each cluster in intestinal stromal cells (IntSCs) of *Lats1/2*$^{iΔ}$-*tdTomato*$^{rPβC}$ mice (KO_) datasets. **c**, Heatmap of top hundred marker gene overlaps between clusters from *Tomato*$^{rPβC}$ and *Lats1/2*$^{iΔ}$-*tdTomato*$^{rPβC}$ mice (KO_). Values are Jaccard similarity coefficient, which is calculated by dividing the number of overlaps by that of the union. **d**, Pie chart visualizing the abundance of PDGFRβ$^+$ cell clusters in each of the four datasets–two replicates of WT and Lats1/2-KO. **e**, Uniform manifold approximation and projection (UMAP) visualization of unsupervised clustering (far left panel) and gene expression level of *Vegfc* projected on UMAP plot (middle panel) in dataset produced by integrating that of *tdTomato*$^{rPβC}$ and *Lats1/2*$^{iΔ}$-*tdTomato*$^{rPβC}$ datasets both at 1 week and 2 weeks after the tamoxifen delivery. Far right panel shows unsupervised clustering of *tdTomato*$^{rPβC}$ and *Lats1/2*$^{iΔ}$-*tdTomato*$^{rPβC}$ mice at 1 week and 2 weeks after the tamoxifen delivery, respectively. Note that all the distinct clusters are transcriptionally conserved between the WT and KO datasets regardless of the time point after the tamoxifen delivery. **f**, Significantly enriched gene ontology (GO) terms of vFB1-3. Selected GO terms that are false discovery rate < 0.05 by Mann-Whitney *U* test are presented. Significance defined as $-\log_{10} Q$ value.

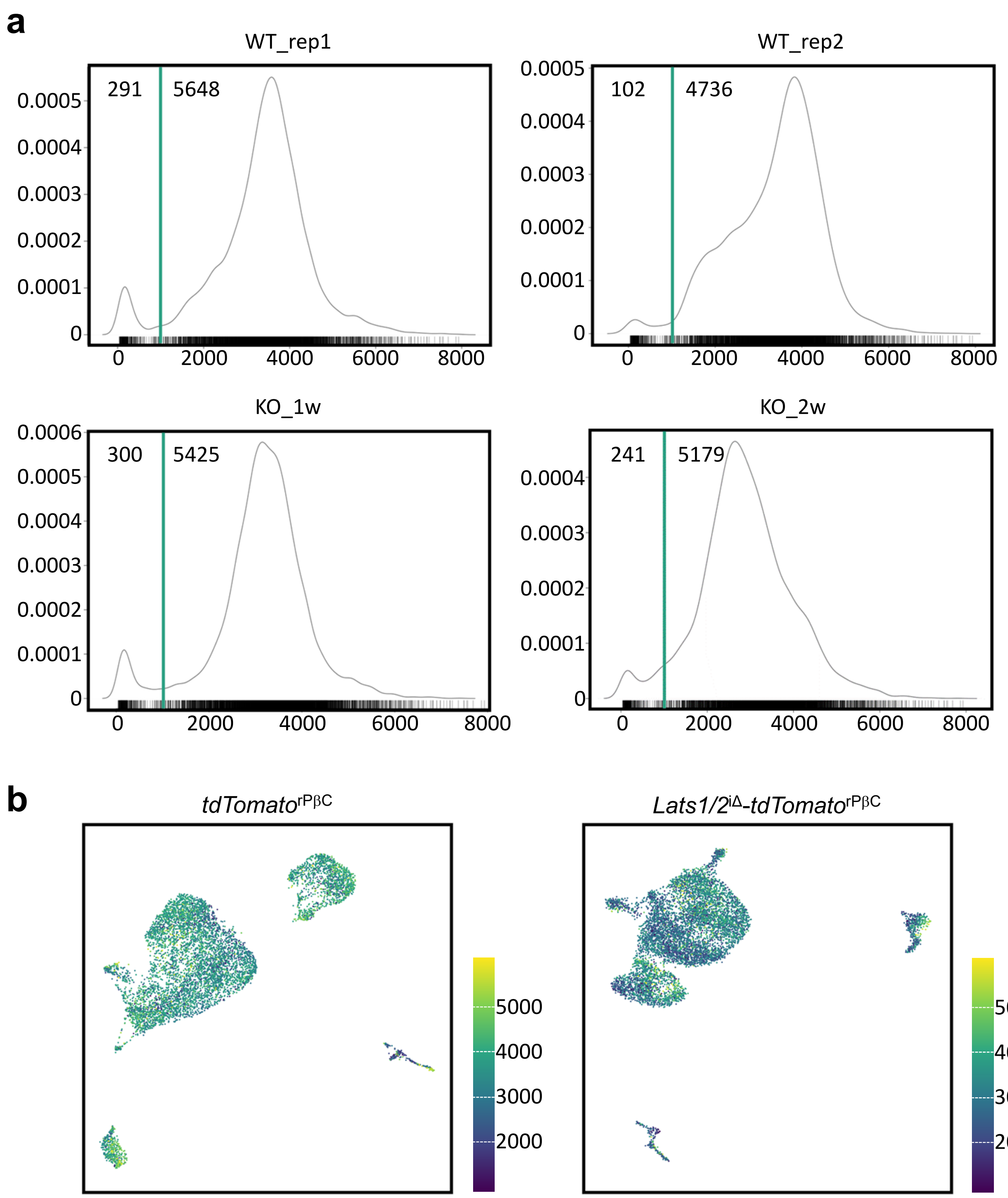


**Supplementary Fig. 17 Cell-level quality control of single-cell RNA sequencing datasets**

**a**, Histogram depicting the distribution of the number of genes detected per cell for each four datasets. The cyan vertical line in the histogram is located at the threshold point (1000 genes). The number in top left corner denotes the number of cells below and above the threshold 1000. **b**, Visualization of number of genes detected per cell in *tdTomato*$^{rP\beta C}$ and *Lats1/2*$^{i\Delta}$-*tdTomato*$^{rP\beta C}$ datasets, projected on the UMAP plot.

## Supplemental Tables

### Supplementary Table 1. Primer sequences for quantitative RT-PCR

| Gene | Primer | Primer sequence (5’ - 3’) |
|---|---|---|
| *Gapdh* | Forward | TGTTCCTACCCCCAATGTGT |
| | Reverse | TGTGAGGGAGATGCTCAGTG |
| *Vegfc* | Forward | CAGGACAGGGGACAGTGTAAAATTTGC |
| | Reverse | TGGCATGCATTGAGTCTTTCTCCAC |
| *Ctgf* | Forward | GTGCCAGAACGCACACTG |
| | Reverse | CCCCGGTTACACTCCAAA |
| *Ankrd1* | Forward | GCTGGTAACAGGCAAAAAGAAC |
| | Reverse | CCTCTCGCAGTTTCTCGCT |
| *Vegfd* | Forward | GGAGAATGGGGAATGGGGAATATCC |
| | Reverse | GTTCAGATCGTTCCAACATGGACCG |
| *Vegfa* | Forward | GCCTCCGAAACCATGAACTTTCTGC |
| | Reverse | CATGGGACTTCTGCTCTCCTTCTG |
| *Ccbe1* | Forward | GGAGACAAATACCCCAATGACACTG |
| | Reverse | CATCTGGGAGAACTCTTTGCATGTG |
| *Adamts3* | Forward | ATTACCTCCTCACCCTGATGAAC |
| | Reverse | ATATGCACTCCGAGGGACTCATC |
| *Tgfb* | Forward | CTGATACGCCTGAGTGGCTGTCTT |
| | Reverse | GAGCAGTGAGCGCTGAATCGAAAG |
| *Ifng* | Forward | CACGGCACAGTCATTGAAAGCCTAG |
| | Reverse | CTTTTGCCAGTTCCTCCAGATATCC |

### Supplementary Table 2. Primer sequences for ChIP-qPCR

| Gene | Primer | Primer sequence (5’ - 3’) |
|---|---|---|
| *Oct4* | Forward | AAAGCGGGTGTCTTATCACTCTG |
| | Reverse | CCTCATCTAACAGACTAAGGGGTTG |
| *Vegfc*_R1 | Forward | GATAATTGGAACTGTCGGGTTAAAC |
| | Reverse | CGTACTTCGTCTAATGTAAATTCCAC |
| *Vegfc*_R2 | Forward | GTTTTACAACCGCCCTTTG |
| | Reverse | CTTCTTTCTGTGTTCCTGTTGAAC |
| *Vegfc*_R3 | Forward | GTGACAGTTCTCAGTGTAGGCTG |
| | Reverse | AGTCACAAAGGGCGGTTG |
