# Supplementary material for: Distinct fibroblast subsets regulate lacteal integrity through YAP/TAZ-induced VEGF-C in intestinal villi
Source: Nat Commun. 2020 Aug 14;11:4102. doi: 10.1038/s41467-020-17886-y (PMC7428020; doi:10.1038/s41467-020-17886-y)
Supplement: Supplementary file 3 — Reporting Summary [file 41467_2020_17886_MOESM3_ESM.pdf]

nature research

Corresponding author(s): Gou Young Koh

Last updated by author(s): Jul 14, 2020

Reporting Summary

Nature Research wishes to improve the reproducibility of the work that we publish. This form provides structure for consistency and transparency in reporting. For further information on Nature Research policies, see Authors & Referees and the Editorial Policy Checklist.

Statistics

For all statistical analyses, confirm that the following items are present in the figure legend, table legend, main text, or Methods section.

n/a	Confirmed	
☐	☒	The exact sample size (n) for each experimental group/condition, given as a discrete number and unit of measurement
☐	☒	A statement on whether measurements were taken from distinct samples or whether the same sample was measured repeatedly
☐	☒	The statistical test(s) used AND whether they are one- or two-sided<br>*Only common tests should be described solely by name; describe more complex techniques in the Methods section.*
☐	☒	A description of all covariates tested
☐	☒	A description of any assumptions or corrections, such as tests of normality and adjustment for multiple comparisons
☐	☒	A full description of the statistical parameters including central tendency (e.g. means) or other basic estimates (e.g. regression coefficient) AND variation (e.g. standard deviation) or associated estimates of uncertainty (e.g. confidence intervals)
☐	☒	For null hypothesis testing, the test statistic (e.g. F, t, r) with confidence intervals, effect sizes, degrees of freedom and P value noted<br>*Give P values as exact values whenever suitable.*
☒	☐	For Bayesian analysis, information on the choice of priors and Markov chain Monte Carlo settings
☒	☐	For hierarchical and complex designs, identification of the appropriate level for tests and full reporting of outcomes
☒	☐	Estimates of effect sizes (e.g. Cohen's d, Pearson's r), indicating how they were calculated

Our web collection on statistics for biologists contains articles on many of the points above.

Software and code

Policy information about availability of computer code

Data collection	The following software were used for data collection:<br>LSM image software (Carl Zeiss)<br>Zen 2.3 software (Carl Zeiss)
Data analysis	The following software were used for data analysis:<br>ImageJ (Fiji version) software (NIH)<br>Zen 2.3 software (Carl Zeiss)<br>Imaris (Bitplane)<br>TopHat software<br>Ingenuity Pathway Analysis tool (QIAGEN)<br>Morpheus (https://software.broadinstitute.org/morpheus/)<br>Cell Ranger 3.0.2 toolkit from 10X Genomics (http://10xgenomics.com)<br>R: The R Project for Statistical Computing<br>R package 'Seurat' (version 3.0.0)<br>R package "goseq" (version 1.34.0)<br>GraphPad Prism 8.0 (GraphPad Software)<br>FlowJo V10 (Treestar)

For manuscripts utilizing custom algorithms or software that are central to the research but not yet described in published literature, software must be made available to editors/reviewers. We strongly encourage code deposition in a community repository (e.g. GitHub). See the Nature Research guidelines for submitting code & software for further information.

Data

Policy information about availability of data

All manuscripts must include a data availability statement. This statement should provide the following information, where applicable:

- Accession codes, unique identifiers, or web links for publicly available datasets
- A list of figures that have associated raw data
- A description of any restrictions on data availability

The bulk RNA-seq data and single cell RNA-seq data are both available in Gene Expression Omnibus (GEO) under the accession number GSE124488. For Gene Set Enrichment Analysis (GSEA), Molecular Signatures Database 4.0 (http://www.broadinstitute.org/gsea/msigdb/) was used. The source data for all figures and supplementary figures are available as Source Data file. Reporting Summary for this article is provided as Supplementary Information file. All the other data and codes used for the analysis are available from the corresponding author upon reasonable request.

Field-specific reporting

Please select the one below that is the best fit for your research. If you are not sure, read the appropriate sections before making your selection.

☒ Life sciences ☐ Behavioural & social sciences ☐ Ecological, evolutionary & environmental sciences

For a reference copy of the document with all sections, see nature.com/documents/nr-reporting-summary-flat.pdf

Life sciences study design

All studies must disclose on these points even when the disclosure is negative.

Sample size	Sample sizes were chosen on the basis of standard power calculations (with α = 0.05 and power of 0.8) performed for similar experiments and statistical methods were not used to predetermine sample sizes as previously published (Robciuc et al., Cell Metabolism, 2016; Shimizu et al., Journal of Clinical Investigation, 2014).
Data exclusions	No samples were excluded from the analysis.
Replication	Experiments were replicated at least once for all analyses and number of reproductions of each experimental finding is described in each figure legend. All attempts at experimental replication were successful.
Randomization	Animals from different cages, but within the same experimental group, were selected to assure randomization. Experiments involving in vitro study was assured randomization through double-blind experiments.
Blinding	The investigators were blinded to allocation during experiments and outcome analyse

Reporting for specific materials, systems and methods

We require information from authors about some types of materials, experimental systems and methods used in many studies. Here, indicate whether each material, system or method listed is relevant to your study. If you are not sure if a list item applies to your research, read the appropriate section before selecting a response.

Materials & experimental systems

n/a	Involved in the study	
☐	☒	Antibodies
☐	☒	Eukaryotic cell lines
☒	☐	Palaeontology
☐	☒	Animals and other organisms
☒	☐	Human research participants
☒	☐	Clinical data

Methods

n/a	Involved in the study	
☒	☐	ChIP-seq
☐	☒	Flow cytometry
☒	☐	MRI-based neuroimaging

Antibodies

Antibodies used	The following primary and secondary antibodies were used in the immunostaining: anti–LYVE-1 (rabbit polyclonal, 11-034, Angiobio, 1:400); anti-CD31 (rat monoclonal, 557355, BD Biosciences, 1:400); anti-CD31 (hamster monoclonal, MAB1398Z, Merck, 1:400); anti-E-cadherin (goat polyclonal, AF748, R&D, 1:200); anti-Prox1 (goat polyclonal, AF2727, R&D, 1:400); anti-Prox1 (rabbit polyclonal, 102-PA32AG, ReliaTech, 1:400); anti-VE-cadherin (goat polyclonal, AF1002, R&D, 1:200); anti-PDGFRβ (rat monoclonal, ab91066, Abcam, 1:200); anti-PAI-1 (mouse monoclonal, sc-5297, Santa Cruz, 1:100); anti-Serpina3n (Goat polyclonal, AF4709, R&D, 1:200); anti-Fosb (rabbit monoclonal, 2251, Cell Signaling Technology, 1:400); anti-Shisa3 (rabbit polyclonal, TA320118, Origene, 1:400); anti-P2X1 (rabbit polyclonal, APR-001, Alomone labs, 1:800); anti-Ackr4 (rabbit

polyclonal, SAB4502137, Sigma-Aldrich, 1:200); anti-Grem1 (goat polyclonal, AF956, R&D, 1:200); anti-Sox6 (rabbit polyclonal, ab30455, Abcam, 1:400); anti-PDGFRα (goat polyclonal, AF1062, R&D, 1:200); anti-YAP (rabbit monoclonal, 14074, Cell signaling, 1:200); anti-TAZ (rabbit polyclonal, HPA007415, Sigma-Aldrich, 1:200); anti-αSMA, Fluorescein Isothiocyanate (FITC)-conjugated (mouse monoclonal, F3777, Sigma-Aldrich, 1:1000); anti-VEGFR3 (goat polyclonal, AF743, R&D, 1:200); anti-VEGFR2 (goat polyclonal, AF644, R&D, 1:200); anti-PGP9.5 (rabbit monoclonal, 13179, Cell signaling, 1:400); anti-F4/80, FITC-conjugated (rat monoclonal, 1231007, Biolegend, 1:200); anti-CD3 (hamster monoclonal, 553058, BD Biosciences, 1:1000); anti-Desmin (rabbit polyclonal, AB907, Millipore, 1:400); and Alexa Fluor 488-, Alexa Fluor 594-, Alexa Fluor 647-conjugated anti-rabbit, anti-rat, anti-goat, anti-hamster secondary antibodies (diluted at a ratio of 1:1000) were purchased from Jackson ImmunoResearch.

For VEGFR2 blockade, we used a VEGFR2 neutralizing antibody (DC101). A hybridoma cell line that produces DC101 was purchased from American Type Culture Collection (ATCC). For control antibody, we used rat IgG-Fc (SAB3700546, Simga-Aldrich).

The following antibodies were used for flow cytometry: FITC anti-mouse CD45 (11-0451-85, rat monoclonal, Biolegend); FITC anti-mouse TER-119 (11-5921-85, rat monoclonal, Biolegend); APC anti-mouse CD31 (551262, rat monoclonal, BD Bioscience); and PE/Cy7 anti-mouse Podoplanin (127412, syrian hamster monoclonal, Biolegend). Blocking Fcγ receptors was performed with mouse anti-CD16/CD32 (553141, BD Bioscience).d

The following primary antibodies were used for the cell staining: anti-YAP (rabbit monoclonal, 14074, Cell signaling); anti-TAZ (rabbit polyclonal, HPA007415, Sigma-Aldrich); and Alexa Fluor 488-conjugated anti-phalloidin (A12379, Thermo Fisher) antibodies. Alexa Fluor 594-conjugated secondary antibodies were purchased from Jackson ImmunoResearch. Nuclei were stained with DAPI (Invitrogen).

Anti-TEAD4 antibody (mouse monoclonal, ab58310, Abcam) was used for ChiP-qPCR.

To enrich the stromal cell fraction and LECs, hematopoietic cells and epithelial cells were depleted using AutoMACS (Miltenyi) after incubation for 15 min on ice with anti-CD45 and anti-CD326 Microbeads (Miltenyi). For IntSC isolation, anti-CD31 Microbeads (Miltenyi) were added to deplete endothelial cells in addition to the anti-CD45 and anti-CD326 Microbeads.

Validation

All the antibodies were validated for the species (mouse or rat) and applications (immunohistochemistry) by the correspondent manufacturer, which is described in the manufacturer's website. Our usage was described in the Methods section of the manuscript as below.
For both whole-mount and section staining, samples were permeabilized and blocked with blocking buffer containing 5% donkey (or goat) serum in 0.3% Triton-X 100 in PBS for 1 h at room temperature (RT). Samples were incubated with the primary antibody diluted in the blocking buffer overnight at 4 °C. After several washes with PBS, samples were incubated for 2 h at RT with the fluorocrome-conjugated secondary antibodies diluted in the blocking buffer. After several washes with PBS, samples were mounted with Vectashield (Vector Laboratories).

Eukaryotic cell lines

Policy information about cell lines

Cell line source(s)

Primary intestinal stromal cells (IntSCs) and lymphatic endothelial cells (LECs) were isolated from the indicated mice as previously described (Stzepourginski I et al., PNAS, 2017).
Mouse embryonic fibroblasts (MEFs) were isolated from E12.5 embryos as previously described (Kim M et al., EMBO, 2013).
Human dermal LECs (HDLECs) were purchased from Lonza.
DC101 hybridoma (ATCC HB-11534; ATCC; rat (B cell), mouse (myeloma); spleen)

Authentication

Primary mouse IntSCs were authenticated as previously described (Stzepourginski I et al., PNAS, 2017), mouse LECs and HDLECs were validated with expression of Prox1. MEFs were isolated and validated as previously described (Kim M et al., EMBO, 2013). Authentications of hybridoma cells were based on morphology, growth conditions, and blocking antibodies purified from these cells lines were tested for specific binding/blocking efficiencies in both in vitro and in vivo settings.

Mycoplasma contamination

All cells were confirmed to be mycoplasma-negative (MycoAlert Detection Kit, Lonza).

Commonly misidentified lines
(See ICLAC register)

None.

Animals and other organisms

Policy information about studies involving animals; ARRIVE guidelines recommended for reporting animal research

Laboratory animals

Pdgfrb-Cre-ERT2 mouse (Kato K et al., Nat Commun, 2018) was provided by Dr. Ralf H Adams (MPI, Germany); Lats1fl/fl/Lats2fl/fl (Cho H et al., Circ Res, 2019) and Yapfl/fl/Tazfl/fl (Cho H et al., Circ Res, 2019) mice were provided by Dr. Daesik Lim (KAIST, Republic of Korea). R26-tdTomato and Myh11-Cre-ERT2 mice were purchased from the Jackson Laboratory. E12.5 embryos were collected from C57BL/6 wild type mice. All mice were maintained in the C57BL/6 background and mice aged 8~12 weeks of both genders were used for experiments.
Mice were housed under 12 light/12 dark cycle, temperatures of 22±2°C with 50±10% humidity.

Wild animals

The study did not involve wild animals.

Field-collected samples

The study did not involve samples collected from the field.

Ethics oversight	All animal care and experimental procedures were complied with all ethical regulations for animal research and testing under the approval from the Institutional Animal Care and Use Committee (No. KA2016-12) of Korea Advanced Institute of Science and Technology (KAIST).

Note that full information on the approval of the study protocol must also be provided in the manuscript.

Flow Cytometry

Plots

Confirm that:

☒ The axis labels state the marker and fluorochrome used (e.g. CD4-FITC).

☒ The axis scales are clearly visible. Include numbers along axes only for bottom left plot of group (a 'group' is an analysis of identical markers).

☒ All plots are contour plots with outliers or pseudocolor plots.

☒ A numerical value for number of cells or percentage (with statistics) is provided.

Methodology

Sample preparation	To sort PDGFRb+ IntSCs or intestinal LECs, the enriched fractions went on RBC lysis by suspension in ACK lysis buffer (Gibco) for 5 min at RT. After blocking Fcγ receptors with mouse anti-CD16/CD32 (553141, BD Bioscience), cells were incubated for 15 min with indicated antibodies in FACS buffer (2% FBS in PBS). After several washes, cells were analyzed by FACS Canto II (BD Biosciences) and the acquired data were further evaluated by using FlowJo software (Treestar). Cell sorting was performed with FACS Aria Fusion (Beckton Dickinson). Dead cells were excluded using DAPI (Sigma-Aldrich) staining and cell doublets were systematically excluded. Fluorescence intensity is expressed in arbitrary units on a logarithmic scale, and forward scatter and side scatter are represented on a linear scale.
Instrument	Analysis was performed using FACS Canto II (BD Biosciences).<br>Sorting was performed using FACS Aria Fusion (Beckton Dickinson).
Software	Analysis was performed using FlowJo V10 software (Treestar).
Cell population abundance	Purity was not assessed.
Gating strategy	For all experiment, cells were gated on single cells, live cells followed by the staining of interest. The gating strategy is indicated above each FACS plot in the figures.

☒ Tick this box to confirm that a figure exemplifying the gating strategy is provided in the Supplementary Information.
